# Loss of SLC27A5 Activates Hepatic Stellate Cells and Promotes Liver Fibrosis via Unconjugated Cholic Acid

**DOI:** 10.1002/advs.202304408

**Published:** 2023-11-13

**Authors:** Kang Wu, Yi Liu, Jie Xia, Jiale Liu, Kai Wang, Huijun Liang, Fengli Xu, Dina Liu, Dan Nie, Xin Tang, Ailong Huang, Chang Chen, Ni Tang

**Affiliations:** ^1^ Key Laboratory of Molecular Biology for Infectious Diseases (Ministry of Education) Institute for Viral Hepatitis Department of Infectious Diseases The Second Affiliated Hospital Chongqing Medical University Chongqing 400010 China; ^2^ Institute of Life Sciences Chongqing Medical University Chongqing 400016 China

**Keywords:** bile acid, early growth response protein 3, hepatic stellate cells, liver fibrosis, solute carrier family 27 member 5

## Abstract

Although the dysregulation of bile acid (BA) composition has been associated with fibrosis progression, its precise roles in liver fibrosis is poorly understood. This study demonstrates that solute carrier family 27 member 5 (SLC27A5), an enzyme involved in BAs metabolism, is substantially downregulated in the liver tissues of patients with cirrhosis and fibrosis mouse models. The downregulation of SLC27A5 depends on RUNX family transcription factor 2 (RUNX2), which serves as a transcriptional repressor. The findings reveal that experimental SLC27A5 knockout (*Slc27a5* *^−/−^*) mice display spontaneous liver fibrosis after 24 months. The loss of SLC27A5 aggravates liver fibrosis induced by carbon tetrachloride (CCI_4_) and thioacetamide (TAA). Mechanistically, SLC27A5 deficiency results in the accumulation of unconjugated BA, particularly cholic acid (CA), in the liver. This accumulation leads to the activation of hepatic stellate cells (HSCs) by upregulated expression of early growth response protein 3 (EGR3). The re‐expression of hepatic SLC27A5 by an adeno‐associated virus or the reduction of CA levels in the liver using A4250, an apical sodium‐dependent bile acid transporter (ASBT) inhibitor, ameliorates liver fibrosis in Slc27a5*^−/−^* mice. In conclusion, SLC27A5 deficiency in mice drives hepatic fibrosis through CA‐induced activation of HSCs, highlighting its significant implications for liver fibrosis treatment.

## Graphical abstract

The authors propose a novel mechanism of aberrant bile acid metabolism in liver fibrosis. Loss of solute carrier family 27 member 5 (SLC27A5) accumulates cholic acids (CA) in the liver and promotes hepatic stellate cells activation via early growth response protein 3 (EGR3). Re‐expression of SLC27A5 or inhibition of CA levels could be a promising strategy for liver fibrosis treatment.

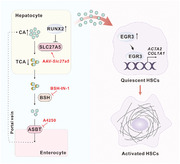

## 1 Introduction

Liver fibrosis is a common feature of chronic liver diseases, associated with advanced disease progression and poor prognosis. Unless effectively treated, fibrosis tends to develop into cirrhosis and subsequently into liver failure, requiring transplantation.^[^ ^1^ ^]^ The underlying etiologies of liver fibrosis include hepatitis B and C infections, alcohol abuse, non‐alcoholic fatty liver disease (NAFLD), and cholestatic injury.^[^ ^2^ ^]^ Hepatic stellate cells (HSCs) play a crucial role in the development of fibrotic processes as they possess the ability to undergo activation and trans‐differentiation into myofibroblast‐like cells. These cells are a primary source of the extracellular matrix (ECM).^[^ ^3^ ^]^ The activation of HSCs involves a complex interaction with other resident cells and their secreted profibrotic mediators, including chemokines, growth factors, reactive oxygen species, and metabolic products.^[^ ^4^ ^]^ Although numerous studies have contributed to our understanding of the pathogenesis of liver fibrosis, the intercellular crosstalk between dysregulated hepatocyte metabolism and HSC activation remains unclear.

Bile acids (BAs) are the end products of cholesterol catabolism in hepatocytes. They are known for liver cholesterol secretion and intestine lipid absorption.^[^ ^5^ ^]^ However, dysregulated BAs homeostasis may result in chronic liver diseases.^[^ ^6^ ^]^ Recent studies have reported increased BAs levels with increasing fibrosis stage.^[^ ^7^ ^]^ The composition of BAs in patients with cirrhosis has been previously reported to be abnormal.^[^ ^8^, ^9^ ^]^ The accumulation of BAs in the liver may lead to hepatocyte mitochondrial injury, cholangiocyte proliferation, or macrophage activation, which result in cholestatic liver injuries and inflammatory responses.^[^ ^10^, ^11^, ^12^ ^]^ Defects in specific genes involved in BAs metabolism contribute to chronic cholestatic liver fibrosis.^[^ ^13^ ^]^

Solute carrier family 27 member 5 (SLC27A5), also known as long‐chain fatty acid transport protein 5 (FATP5), is mainly expressed in the liver, specifically in the basement membrane of hepatocytes, where it participates in fatty acid transport.^[^ ^14^ ^]^ SLC27A5 exhibits bile acid‐CoA ligase (BAL) activity, which converts unconjugated BAs to their CoA thioester derivatives and catalyzes the conjugation of BAs with amino acids.^[^ ^15^, ^16^ ^]^ Mice lacking SLC27A5 have shown to exhibit higher levels of unconjugated BAs and lower levels of conjugated BAs, as compared to normal mice.^[^ ^16^, ^17^ ^]^ This is consistent with elevated unconjugated BAs observed in patients with genetic defects in SLC27A5.^[^ ^18^, ^19^ ^]^ Silencing of SLC27A5 in mice corresponds to the high proportion of unconjugated BAs reported in these patients. Notably, a neonate with a homozygotic missense mutation in SLC27A5 was found to develop extensive liver fibrosis.^[^ ^18^ ^]^ Additionally, Enooku et al. demonstrated that lower SLC27A5 expression is associated with the progression of ballooning and fibrosis in patients with NAFLD.^[^ ^20^ ^]^ However, the specific mechanisms underlying SLC27A5 deficiency in liver fibrosis remain unclear.

Therefore, we aimed to elucidate the role of SLC27A5 in regulating BAs conjugation during the progression of liver fibrosis. Furthermore, we monitored the effect of the lack of SLC27A5 in mice on the activation of HSCs and liver fibrosis and investigated the associated molecular mechanisms. We believe that our findings can provide mechanistic insights into the role of SLC27A5 in regulating liver fibrosis.

## 2 Results

### 2.1 SLC27A5 Expression is Downregulated in Human and Murine Liver Fibrosis

**Figure 1:**
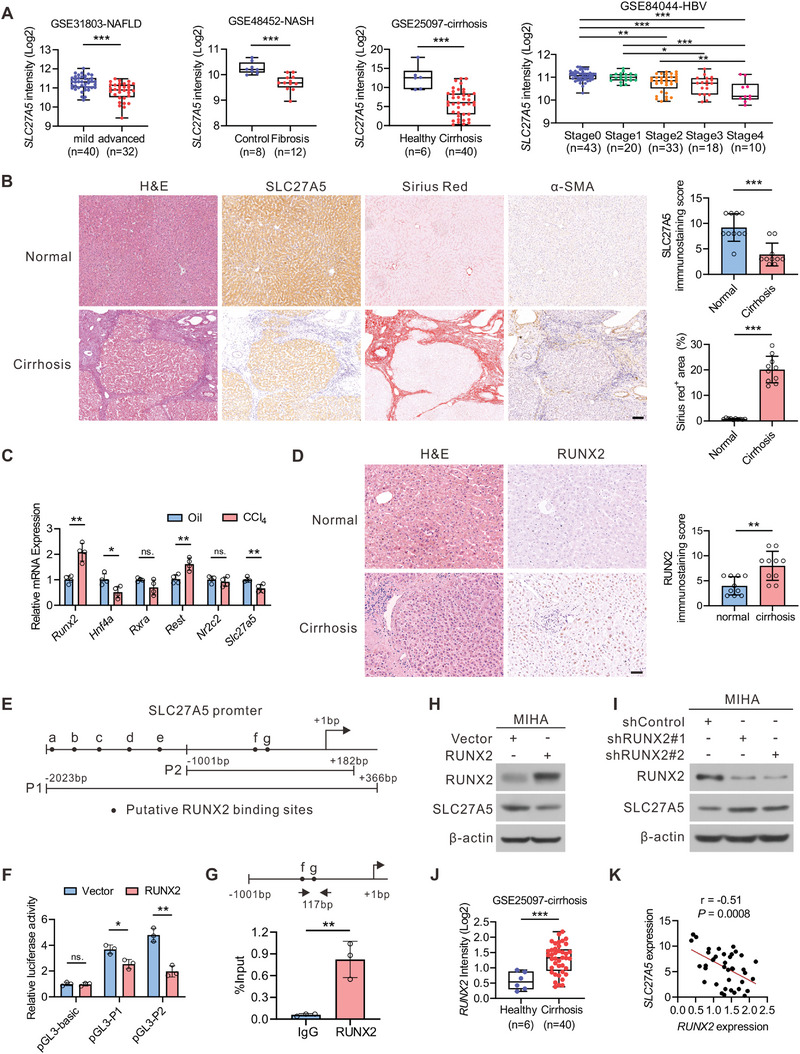
SLC27A5 expression is generally down‐regulated in the livers of cirrhosis patients and CCI_4_‐treated mice A) Box plots of relative *SLC27A5* mRNA levels in GSE31803, GSE48452, GSE25097, and GSE84044 datasets. B) Representative liver histology of H&E, Sirius Red staining, SLC27A5, and α‐SMA IHC in consecutive sections of normal and cirrhotic human livers, and its statistical summary (n = 10 per group). Scale bar, 100 µm. C) The *Runx2*, *Hnf4a*, *Rxra*, *Rest*, and *Nr2c2* expression levels were detected using qRT‐PCR in WT mice livers subjected to CCl_4_ treatment (n = 4 per group). D) Representative H&E and RUNX2 IHC in liver sections from healthy controls and patients with cirrhosis, and its statistical summary (n = 10 per group). Scale bar: 50 µm. E) Putative binding sites of RUNX2 (black spots) in the *SLC27A5* gene promoter (−2023/+366). F) MIHA cells were co‐transfected with the *SLC27A5* promoter luciferase reporter and expression plasmids for RUNX2, and the luciferase activity was monitored as described in the panel (n = 3 per group). G) ChIP‐qPCR analysis to determine the binding of RUNX2 protein to the SLC27A5 promoter in MIHA cells. Diagram of the SLC27A5 gene promoter (−1001/+182) depicting the location of the amplified region (−396/−280) (n = 3 per group). H) MIHA cells were transfected with vector or RUNX2‐overexpressing plasmid. The protein levels of RUNX2 and SLC27A5 were analyzed using Western blotting. I) MIHA cells were transfected with shControl or shRUNX2. The expression of RUNX2 and SLC27A5 was detected. J) Box plots of relative mRNA levels of *RUNX2* in the GSE25097 dataset. K) Correlation of hepatic *RUNX2* mRNA with *SLC27A5* in patients with cirrhosis (n = 40 for GSE25097). Data are presented as mean ± SEM. *P < 0.05, **P < 0.01, ***P < 0.001, ns., not significant. Data in (A) (left three panels), (B–D), (F–G), and (J) were analyzed using two‐tailed unpaired Student's *t*‐test. One‐way ANOVA analyzed data in (A) (right panel) with Tukey's post hoc test. Data in (K) was analyzed using Pearson correlation coefficient analysis.

To evaluate the role of SLC27A5 in the development of liver fibrosis, we analyzed *SLC27A5* mRNA expression levels in normal and fibrotic liver biopsy samples from the Gene Expression Omnibus (GEO) database. We observed downregulation of hepatic *SLC27A5* mRNA expression in patients with NAFLD coupled with fibrosis, non‐alcoholic steatohepatitis (NASH) with fibrosis, and cirrhosis, as displayed in **Figure** **1**A. We also observed a stepwise decrease in the level of SLC27A5 transcript from fibrosis stages 0 to 4 in a cohort of patients with hepatitis B virus (HBV)‐related liver fibrosis (Figure 1A). Analyzing the cirrhosis dataset (GSE25097) revealed that the expression of SLC27A5 was negatively correlated with that of fibrosis‐related genes such as *ACTA2*, *COL1A1*, and *COL3A1* (Figure S1A, Supporting Information). Consistently, human cirrhotic tissue samples (Figure 1B; Figure S1B,C, Supporting Information) and mouse models of liver fibrosis induced by carbon tetrachloride (CCI_4_) and thioacetamide (TAA) (Figure S1D–I, Supporting Information) also revealed reduced SLC27A5 expression compared with that in the control trials.

To identify the putative transcription factors (TFs) responsible for the downregulation of SLC27A5 in liver fibrosis, we analyzed the potential promoter region of *SLC27A5* using the JASPAR database considering the 2‐kb upstream sequence of the transcription start site of *SLC27A5*.^[^ ^21^ ^]^ Further analysis helped identify five TFs (NR2C2, REST, RXRA, HNF4α, and RUNX2) that may bind to the *SLC27A5* promoter region (Figure S2A,B, Supporting Information). Notably, qRT‐PCR demonstrated a significant upregulation of *Runx2* mRNA levels in CCI_4_‐induced fibrotic liver tissues of mice, whereas *Hnf4a* and *Rest* indicated only minor changes (Figure 1C). Additionally, substantially elevated RUNX2 expression was observed in the liver tissues of patients with cirrhosis and fibrosis model mice (Figure 1D; Figure S2C–F, Supporting Information).

Subsequently, we investigated whether RUNX2 was involved in the transcriptional regulation of SLC27A5. According to the prediction made using JASPAR, RUNX2 may bind the promoter of *SLC27A5* at several possible sites (Figure 1E). To determine regulatory regions of RUNX2, the *SLC27A5* promoter regions containing different RUNX2 binding sites were cloned into the pGL3 basic vector to perform luciferase reporter assay. We truncated the full‐length region of the *SLC27A5* promoter into two fragments, −2023/+366 (pGL3‐P1) and −1001/+182 (pGL3‐P2) (Figure 1E). Notably, RUNX2 substantially decreased the activity of both *SLC27A5* promoter fragments in the normal human liver cell line MIHA, which further indicates that SLC27A5 is transcriptionally inhibited by RUNX2 (Figure 1F). Chromatin immunoprecipitation assay confirmed the binding of RUNX2 to the *SLC27A5* promoter (Figure 1G). RUNX2 overexpression significantly downregulated SLC27A5 expression in MIHA cells (Figure 1H), whereas the inhibition of RUNX2 by shRNA led to elevated expression of SLC27A5 (Figure 1I). These findings suggest that RUNX2 is responsible for the downregulation of SLC27A5.

The upregulation of RUNX2 was verified in the cirrhosis, NAFLD, NASH and HBV‐related fibrosis datasets (Figure 1J; Figure S2G–I, Supporting Information). *RUNX2* expression was negatively correlated with *SLC27A5* mRNA levels in cirrhotic tissues (GSE25097) (Figure 1K). These results indicate that the elevated expression of the repressor RUNX2 impairs SLC27A5 transcription in patients with liver fibrosis and mouse models.

### 2.2 SLC27A5 Deficiency Induces Spontaneous Hepatic Fibrosis in 24‐Month‐Old Mice

We used the CRISPR‐Cas9 system to generate whole‐body SLC27A5 knockout (*Slc27a5* ^−/−^) mice by deleting the protein‐coding exons of *Slc27a5* (Figure S3A, Supporting Information) to investigate the loss‐of‐function effect of SLC27A5 in vivo. The genotypes of the mice were determined through PCR analysis of tail DNA (Figure S3B, Supporting Information). The serum ALT, AST and ALP levels showed a mild increase in *Slc27a5* ^−/−^ mice at 12 months compared to WT littermates (Figure S3C, Supporting Information). The mRNA levels of inflammatory genes (*Tnfa*, *Il6*, *Il1b*, *Ccl2*, *Adgre1*) were substantially upregulated in the livers of 12‐month‐old *Slc27a5* ^−/−^ mice (Figure S3D, Supporting Information). These results indicated that *Slc27a5* ^−/−^ mice developed liver injury and inflammatory response at 12 months of age.

**Figure 2:**
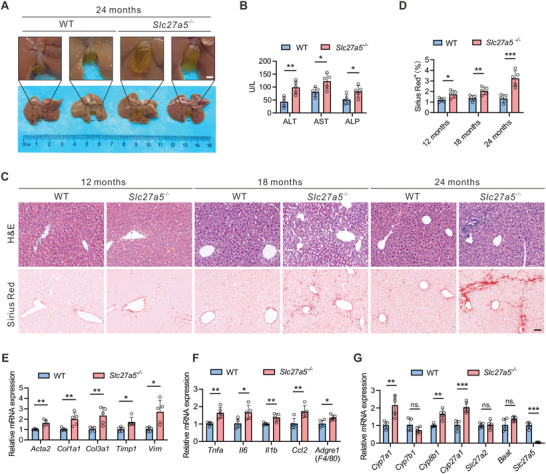
SLC27A5 deficiency in mice develops liver fibrosis after 24 months of age A) Representative pictures of livers from 24‐month‐old WT and *Slc27a5* ^−/−^ mice. Scale bar, 2 mm. B) Serum ALT, AST, and ALP levels from 24‐month‐old WT and *Slc27a5* ^−/−^ mice (n  =  5 per group). C) Representative images of H&E and Sirius Red staining from liver tissues of WT and *Slc27a5* ^−/−^ mice at 12, 18, and 24 months (n  =  5 per group). Scale bar, 50 µm. D) Quantification of Sirius red staining is described in (C). E,F) Relative mRNA levels of profibrotic genes (E) and inflammatory genes (F) of liver tissues from 24‐month‐old WT and *Slc27a5* ^−/−^ mice (n  =  5 per group). G) The hepatic mRNA levels of gene expression of bile acids synthesis in 24‐month‐old WT and *Slc27a5* ^−/−^ mice (n  =  5 per group). Data are presented as mean ± SEM. *P < 0.05, **P < 0.01, ***P < 0.001, ns., not significant, two‐tailed Student's *t* test.

We then maintained wild‐type (WT) and *Slc27a5* ^−/−^ mice for 24 months and observed an increase in the size of gallbladders in *Slc27a5* ^−/−^ mice (**Figure** **2**A). *SLC27A5* ^−/−^ mice exhibited a significant increase in the liver‐to‐body weight ratio and elevated serum levels of alanine aminotransferase (ALT), aspartate aminotransferase (AST), alkaline phosphatase (ALP), and total bilirubin (TBil) compared with that in WT littermates (Figure 2B; Figure S3E,F, Supporting Information). Histological analysis using H&E and Sirius red staining revealed mild collagen deposition in *Slc27a5* ^−/−^ mice at 12 months old (Figure 2C,D). With aging, *Slc27a5* ^−/−^ mice displayed a massive diffuse ECM and pseudo‐lobular nodule formation at 18 and 24 months, respectively (Figure 2C,D). We observed that mRNA levels of profibrotic genes (*Acta2*, *Col1a1*, *Col3a1*, *Timp1*, and *Vim*) and inflammatory genes were substantially upregulated in the livers of 24‐month‐old *Slc27a5* ^−/−^ mice (Figure 2E,F). Liver hydroxyproline (HYP) levels were also increased in *Slc27a5* ^−/−^ mice (Figure S3G, Supporting Information). Western blotting analysis demonstrated a significant increase in the expression of activated HSCs marker α‐SMA and collagen deposition markers COL1A1 and COL3A1 in *Slc27a5* ^−/−^ mice liver tissues (Figure S3H, Supporting Information). Furthermore, the expression levels of hepatic genes involved in bile acid synthesis, including *Cyp7a1*, *Cyp8b1*, and *Cyp27a1*, were significantly increased in *Slc27a5* ^−/−^ mice (Figure 2G). These findings demonstrate that *Slc27a5* deficiency in mice induces spontaneous liver fibrosis at 24 months of age.

### 2.3 SLC27A5 Deficiency Promotes Liver Fibrosis in Chemical‐Induced Fibrosis Murine Models

**Figure 3:**
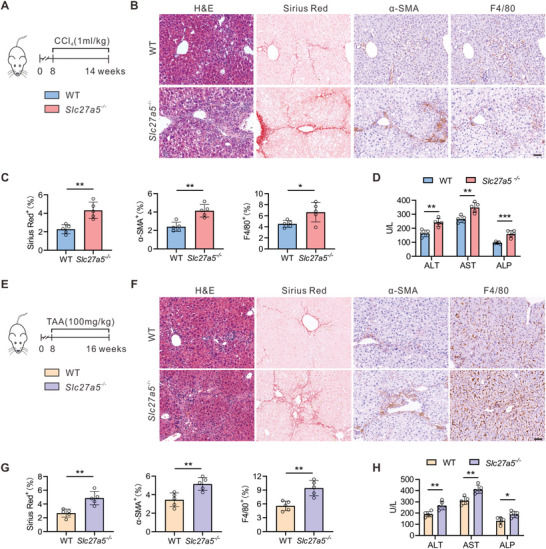
SLC27A5 deficiency promotes CCl_4_‐ and TAA‐induced liver fibrosis in mice A–D) Eight‐week‐old male WT and *Slc27a5* ^−/−^ mice were injected with CCl_4_ for six weeks (n  =  5 per group). E–H) Eight‐week‐old male WT and *Slc27a5* ^−/−^ mice were injected with TAA for eight weeks (n  =  5 per group). (A,E) The experimental approach of the animal model establishment. (B,F) Representative histology of H&E, Sirius Red, and IHC staining (α‐SMA, F4/80) from each group. Scale bar, 50 µm. (C) Quantification of Sirius red, F4/80, and α‐SMA IHC staining are described in (B). G) Quantification of Sirius red, F4/80, and α‐SMA IHC staining are described in (F). (D,H) Serum levels of ALT, AST, and ALP were measured. Data are presented as mean ± SEM. *P < 0.05, **P < 0.01, ***P < 0.001, two‐tailed Student's *t* test.

To further examine the role of SLC27A5 in liver fibrosis, we investigated fibrogenesis in *Slc27a5* ^−/−^ mice subjected to CCl_4_ and TAA treatment. We observed an increase in the liver‐to‐body weight ratio in *Slc27a5* *^−/−^* mice after six weeks of CCl_4_ treatment (**Figure** **3**A; Figure S4A, Supporting Information). Furthermore, H&E, Sirius red staining, α‐SMA, and F4/80 immunostaining revealed elevated collagen deposition, fibrogenesis, and macrophage infiltration in *Slc27a5* *^−/−^* mice (Figure 3B,C). Hydroxyproline measurements confirmed increased collagen deposition in *Slc27a5* *^−/−^* mice (Figure S4B, Supporting Information). Compared with WT mice, *Slc27a5* *^−/−^* mice displayed significantly higher levels of serum ALT, AST, ALP, and total bilirubin, indicating increased liver injury (Figure 3D; Figure S4C, Supporting Information). The upregulation of profibrotic and inflammatory genes were also observed in the livers of *Slc27a5* *^−/−^* mice (Figure S4D–F, Supporting Information). Eight weeks after TAA injection (Figure 3E), *Slc27a5* *^−/−^* mice demonstrated increased collagen deposition, α‐SMA expression, and liver injury (Figure 3F–H; Figure S4G–I, Supporting Information). Similar to the CCl_4_ treatment, the hepatic expression of profibrotic and inflammatory genes increased in *Slc27a5* *^−/−^* mice compared with that in WT controls (Figure S4J–L, Supporting Information). These findings collectively suggest that SLC27A5 deficiency exacerbates liver fibrosis in mouse models.

**Figure 4:**
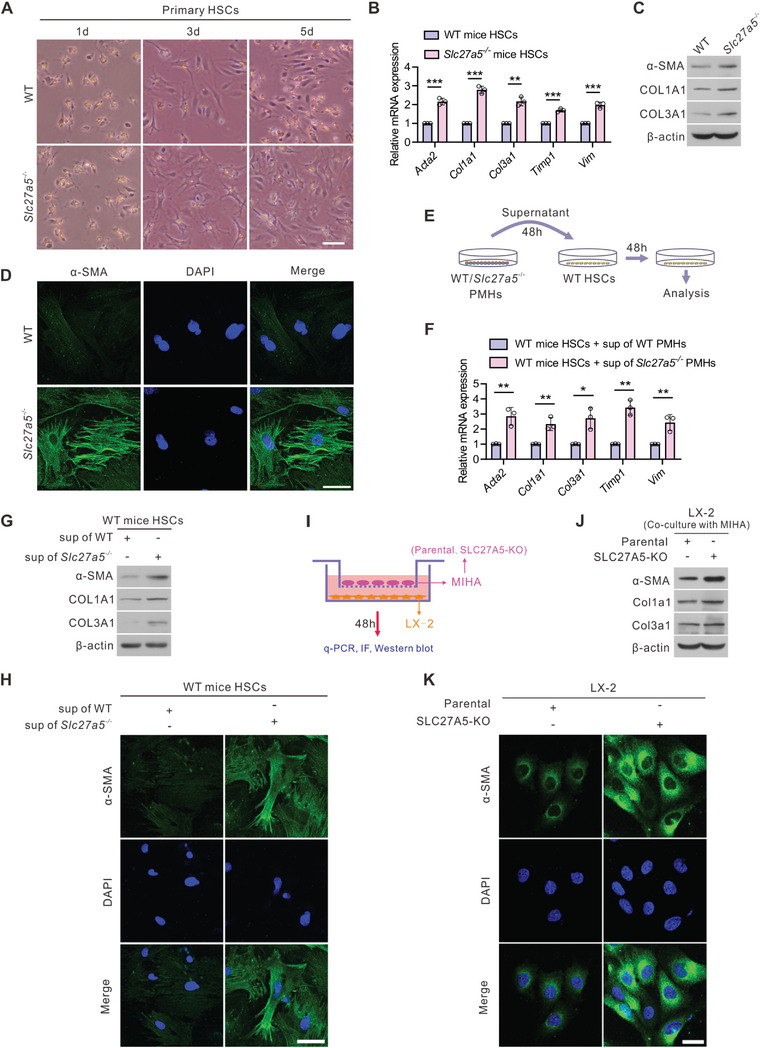
SLC27A5 loss in hepatocytes promotes HSCs activation in vitro. A) Primary mouse HSCs were isolated from WT and *Slc27a5* ^−/−^ mice and culture‐activated for the indicated duration. Cell morphology as observed under light‐field microscopy. Scale bar: 50 µm. B–D) Primary HSCs isolated from WT and *Slc27a5* ^−/−^ mice were culture‐activated for 72 h (n = 3 per group). mRNA expression of fibrogenic genes (B), protein expression of α‐SMA, COL1A1, and COL3A1 (C), and immunofluorescence of α‐SMA (D) are displayed. Scale bar, 25 µm. DAPI, 4′,6‐diamidino‐2‐phenylindole. E) Schematic of co‐culture experiments. F–H) Primary HSCs from WT mice were incubated with the supernatant from WT or *Slc27a5* ^−/−^ PMHs for 48 h. mRNA expression of fibrogenic genes (F), protein expression of α‐SMA, COL1A1, and COL3A1 (G), and immunofluorescence of α‐SMA (H) are displayed. Scale bar, 25 µm. I) Schematic flow chart of co‐culture models. (J‐K) LX‐2 cells were co‐cultured with parental and SLC27A5‐KO MIHA cells for 48 h. Protein expression of α‐SMA, COL1A1, and COL3A1 J), and immunofluorescence of α‐SMA K) are displayed. Scale bar, 25 µm. Data are presented as mean ± SEM. *P < 0.05, **P < 0.01, ***P < 0.001, two‐tailed Student's *t* test.

### 2.4 SLC27A5 Loss in Hepatocytes Promotes HSC Activation

We compared the primary HSCs isolated from *Slc27a5* ^−/−^ and WT mice to assess their culture‐induced activation. The HSCs from *Slc27a5* ^−/−^ mice exhibited enhanced activation, as evidenced by changes in cell morphology, loss of lipid droplets, and a myofibroblast‐like appearance (**Figure** **4**A). The expression of fibrogenic marker genes was induced in these cells (Figure 4B–D). We further determined the sensitivity of *Slc27a5* ^−/−^ HSCs to the transforming growth factor β1 (TGFβ1), which is regarded as a critical factor in the HSCs activation. The primary HSCs derived from *Slc27a5* ^−/−^ mice displayed increased sensitivity to TGFβ1‐stimulated activation compared with that of WT HSCs (Figure S5A–C, Supporting Information). These findings suggest that primary HSCs from *Slc27a5* ^−/−^ mice exhibit enhanced culture‐induced activation and increased sensitivity to TGFβ1 stimulation in vitro.

The crosstalk between hepatocytes and HSCs generates a permissive fibrotic microenvironment that contributes to fibrosis.^[^ ^4^ ^]^ Given that SLC27A5 mainly functions in hepatocytes,^[^ ^14^ ^]^ we first examined the expression of SLC27A5 in primary mouse hepatocytes (PMHs) and primary HSCs of WT mice, and observed that SLC27A5 was only expressed in PMHs but not in primary HSCs (Figure S5D, Supporting Information). We next investigated the contribution of *Slc27a5* ^−/−^ PMHs to fibrotic progression. The WT or *Slc27a5* ^−/−^ PMH supernatant was added to a primary culture of WT mice HSCs for 48 h (Figure 4E). Our results demonstrated a significant up‐regulation of fibrogenic markers in WT HSCs exposed to *Slc27a5* ^−/−^ PMH supernatant (Figure 4F–H). Next, we performed a co‐culture experiment using a combination of the human HSC cell line LX‐2 and MIHA cells in a contact‐independent manner (Figure 4I). The increased expression of fibrogenic genes in LX‐2 cells co‐cultured with SLC27A5‐KO MIHA cells was similar to those observed earlier in this study (Figure 4J,K; Figure S5E,F, Supporting Information). These findings indicate that the loss of SLC27A5 in hepatocytes contributes to HSC activation and increased sensitivity to fibrosis.

### 2.5 Elevated Unconjugated Cholic Acid (CA) Activates HSCs in *Slc27a5* ^−/−^ Mouse

**Figure 5:**
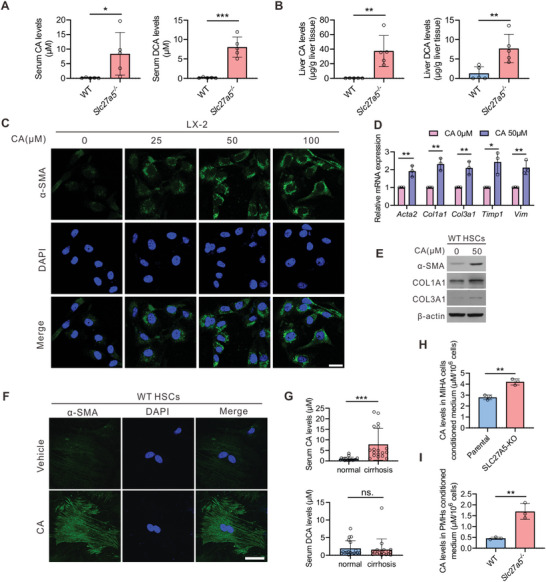
Elevation of unconjugated CA promotes HSCs activation. A) Serum CA and DCA levels in *Slc27a5* ^−/−^ and WT mice at 24 months of age (n = 5 per group). B) Liver CA and DCA levels in *Slc27a5* ^−/−^ and WT mice at 24 months of age (n = 5 per group). C) LX‐2 cells were treated with CA at 25, 50, and 100 µм for 48 h. The expression of α‐SMA was measured using immunostaining. D–F) WT mice HSCs were treated with CA at 50 µм for 48 h. mRNA expression of fibrogenic genes (D), protein expression of α‐SMA, COL1A1, and COL3A1 (E), and α‐SMA immunostaining (F) are displayed. G) The serum levels of CA and DCA in healthy individuals and patients with cirrhosis (n = 18 per group). H) CA levels were measured in media from parental and SLC27A5‐KO MIHA cells (n = 3 per group). I) CA levels were measured in PMHs supernatant from WT and *Slc27a5* ^−/−^ mice (n = 3 per group). Data are presented as mean ± SEM. *P < 0.05, **P < 0.01, ***P < 0.001, two‐tailed Student's *t* test.

SLC27A5 is required for bile acid conjugation and plays an essential role in regulating BAs homeostasis in the liver.^[^ ^22^ ^]^ In this study, we observed an increase in unconjugated BAs, including cholic acid (CA), deoxycholic acid (DCA), and muricholic acid (MCA) in 6‐month‐old *Slc27a5* ^−/−^ mice liver tissues and serum (Figure S6A–D, Supporting Information). Furthermore, analysis of the bile acid composition of 24‐month‐old *Slc27a5* ^−/−^ mice revealed a significant increase in CA and DCA (**Figure** **5**A,B), two major bile acids present in both mice and humans. The reported range of BA levels in human portal venous plasma or serum is 9—43 µм.^[^ ^23^ ^]^ To determine whether the elevated BAs in *Slc27a5* ^−/−^ mice contribute to the activation of HSCs, we treated human HSC cell line LX‐2 with CA or DCA at concentrations of 25, 50, and 100 µм. Interestingly, CA treatment upregulated the expression of HSCs activation markers in a dose‐dependent manner (Figure 5C; Figure S6E,F, Supporting Information). This was further confirmed in primary HSCs from WT mice treated with CA at 50 µм. (Figure 5D–F). However, DCA supplementation did not strongly affect LX‐2 activation (Figure S6G–H, Supporting Information). The mRNA and protein expression of fibrogenic genes were also increased in primary HSCs from *Slc27a5* ^−/−^ mice after CA treatment (Figure S6I,J, Supporting Information). Furthermore, the serum levels of CA, but not DCA, were significantly increased in patients with cirrhosis (Figure 5G). Consistent with these, CA levels were also higher in SLC27A5‐KO MIHA cells and primary mouse hepatocytes (PMHs) compared with that in controls (Figure 5H,I). Based on these observations, we concluded that the abnormal elevation of unconjugated CA in mouse models and patients with cirrhosis promotes HSC activation.

To determine whether the inhibition of CA levels could diminish the activation of HSCs induced by the supernatant of *Slc27a5* ^−/−^ PMHs, WT and *Slc27a5* ^−/−^ mice were treated with BSH‐IN‐1, a covalent pan‐inhibitor of gut bacterial bile salt hydrolases (BSHs),^[^ ^24^ ^]^ which decreased unconjugated bile acids (especially CA in Slc27a5^−/−^ mice) in vivo (Figure S7A, Supporting Information). Then, the supernatant was added to HSCs from normal WT mice (Figure S7B, Supporting Information). The results suggested that the expression of profibrotic genes in primary HSCs was decreased by the treatment of conditional medium from primary PMHs of *Slc27a5* ^−/−^ mice with BSH‐IN‐1 gavage (Figure S7C, Supporting Information). The CA levels were also decreased in the conditional medium of BSH‐IN‐1 group, whereas the TCA levels were increased in the same medium (Figure S7D,E, Supporting Information). Taken together, these results indicate that inhibition of unconjugated CA levels could decrease the expression of profibrotic genes in primary HSCs co‐cultured with the supernatant of *Slc27a5* ^−/−^ PMHs.

### 2.6 Cholic Acid‐Triggered Upregulation of Early Growth Response (EGR3) Promotes HSC Activation

**Figure 6:**
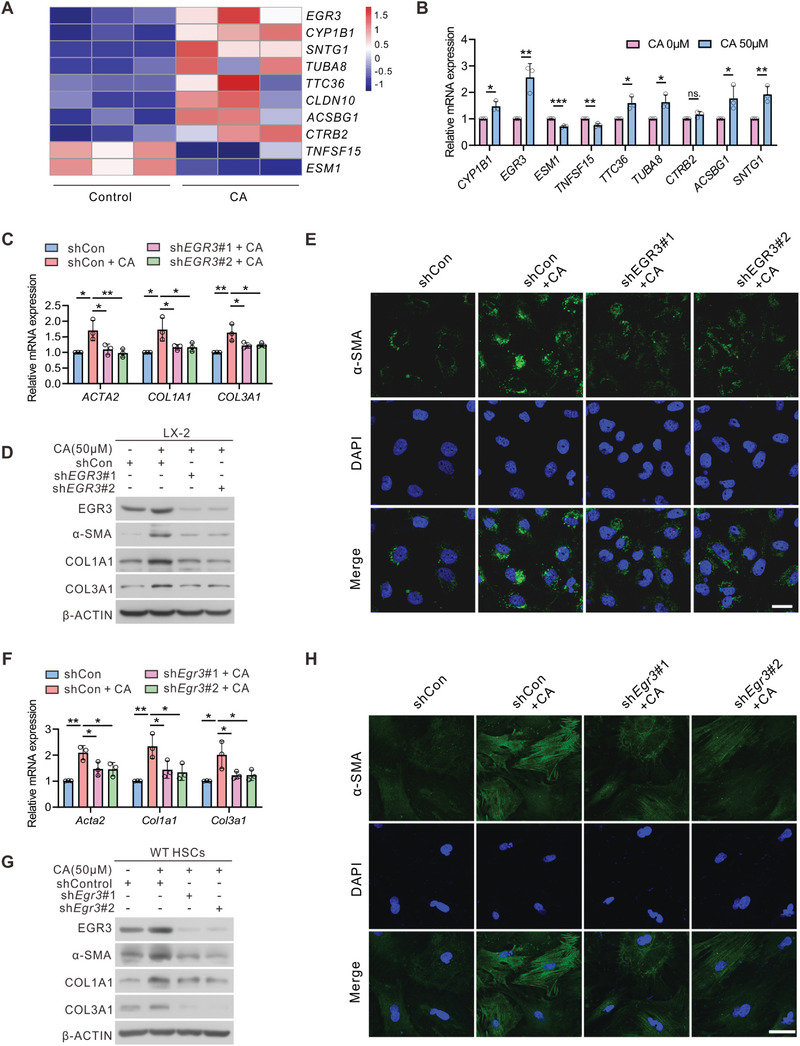
CA‐triggered activation of HSC is dependent on EGR3. A) Expression profile of regulatory genes of fibrosis in LX‐2 cells treated with 50 µм CA or vehicle controls based on RNA‐seq data (n = 3 per group). B) qRT‐PCR was performed to validate the expression of fibrosis‐regulated genes in vehicle‐ and CA‐treated LX‐2 cells (n = 3 per group). C–E) LX‐2 cells were transfected with Control (shCon) or shEGR3 plasmid in the presence of 50 µм CA for 48 h. Expression of the fibrotic genes was analyzed using qRT‐PCR (C), Western blotting (D), or immunofluorescence (E). Scale bar, 25 µm. F–H) Primary mouse HSCs were isolated from WT mice and transfected with Control (shCon) or shEgr3 plasmid in 50 µм CA for 48 h. Expression of the fibrotic genes was analyzed using qRT‐PCR (F), Western blotting (G), or immunofluorescence (H). Scale bar, 25 µm. Data are presented as mean ± SEM. *P < 0.05, **P < 0.01, ***P < 0.001, ns., not significant. Two‐tailed unpaired Student's *t* test was used to analyze data in (B). Data in (C) and (F) were analyzed using one‐way ANOVA with Tukey's post hoc test.

To investigate how CA induced the activation of HSCs, we performed RNA‐seq analysis on LX‐2 cells treated with CA at a concentration of 50 µм or vehicle controls. The treatment with CA effectively increased the expression of regulatory genes associated with fibrosis in LX‐2 cells (**Figure** **6**A). Among the upregulated genes in LX‐2 cells, we observed the most significant upregulation in the expression of pro‐fibrotic transcription factor EGR3 following CA treatment (Figure 6B). However, taurodeoxycholate (TDCA), DCA, or taurocholic acid (TCA) supplementation did not significantly affect EGR3 expression (Figure S8A, Supporting Information). EGR3 is upregulated in the fibrotic dermis of mice with scleroderma, and increased EGR3 expression contributes to the upregulation of fibrotic genes such as *ACTA2* and *COL1A1*.^[^ ^25^ ^]^ To determine the role of EGR3 in CA‐induced activation of HSCs, we performed ChIP assay to confirm the binding of EGR3 on the promoter of *ACTA2* and *COL1A1* gene in LX‐2 cells. The data indicated that EGR3 could bind the promoter of *ACTA2* and *COL1A1* directly, and the recruitment of EGR3 on *ACTA2* and *COL1A1* promoter was increased by CA stimulation (Figure S8B, Supporting Information). Based on these results, we investigated whether CA triggers HSC activation in an EGR3‐dependent manner. Treatment of LX‐2 cells with CA induced the expression of fibrotic genes. However, the induction of these genes was significantly compromised by EGR3 silencing (Figure 6C,D). Furthermore, immunofluorescence assays demonstrated that sh*EGR3* treatment attenuated the CA‐induced expression of α‐SMA (Figure 6E). Notably, the upregulation of α‐SMA, COL1A1, and COL3A1 was similarly compromised by EGR3 silencing in primary mouse HSCs (Figure 6F–H). We also utilized the MIHA and LX‐2 cells to explore the role of EGR3. As displayed in Figure S8B–D (Supporting Information), EGR3 silencing inhibited LX‐2 cell activation induced by SLC27A5‐KO MIHA cell co‐culture. Our findings thus indicate that CA‐induced HSCs activation depends on the transcription factor EGR3.

### 2.7 Overexpression of SLC27A5 or Inhibition of Intestinal Bile Acid Absorption Ameliorates CCl_4_‐Induced Liver Fibrosis

**Figure 7:**
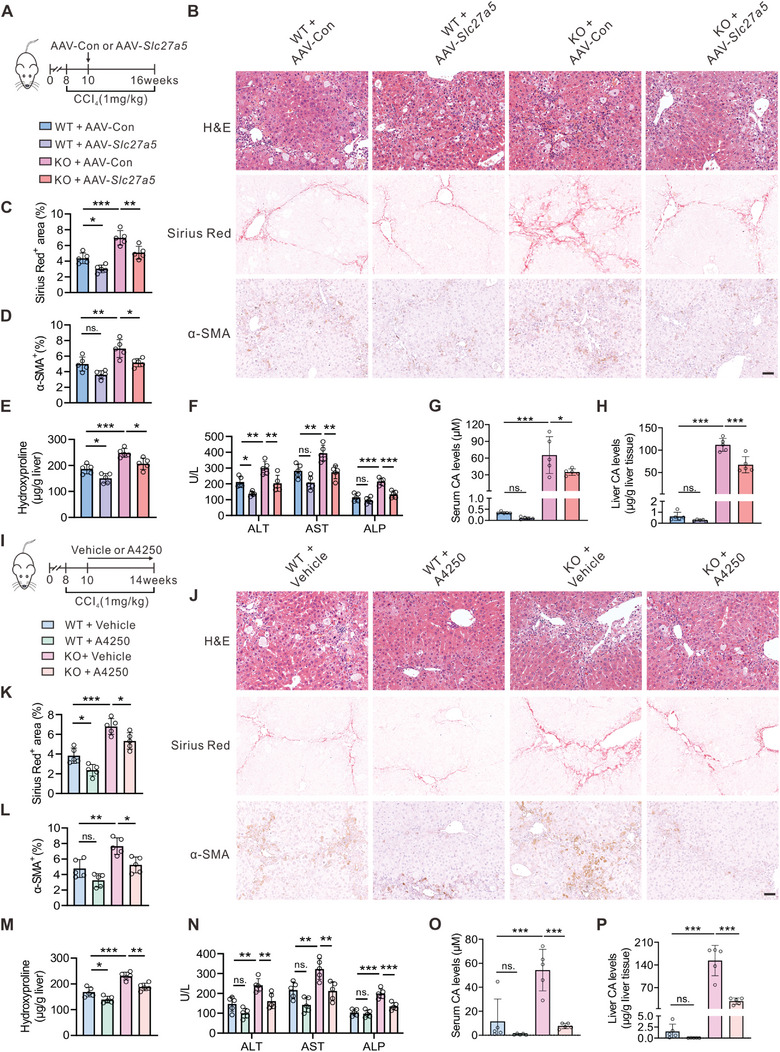
Overexpression of SLC27A5 or inhibition of intestinal bile acid absorption ameliorates CCl_4_‐induced liver fibrosis A–H) Eight‐week‐old male WT and *Slc27a5* *^−/−^* (KO) mice were subjected to CCl_4_ treatment and injected with AAV‐Control (AAV‐Con) or AAV‐*Slc27a5* (n = 5 per group). I–P) Eight‐week‐old male WT and *Slc27a5* *^−/−^* (KO) mice were subjected to CCl_4_ model and treated with vehicle or A4250 by daily gavages as outlined in (I) (n = 5 per group). (A,I) The experimental approach of the animal model establishment. (B,J) Representative liver histology of H&E, Sirius Red, and α‐SMA IHC staining. Scale Bar, 50 µm. (C,D,K,L) Quantification of Sirius red (C,K) and α‐SMA IHC (D,L) staining described in (B,J). (E,M) Hepatic hydroxyproline content was measured. (F,N) Serum levels of ALT, AST, and ALP were measured. (G,H,O,P) CA levels in serum (G,O) and liver (H,P). Data are presented as mean ± SEM. *P < 0.05, **P < 0.01, ***P < 0.001, ns., not significant, one‐way ANOVA with Tukey's post hoc test.

Based on our observations, we observed that SLC2A75 expression was downregulated in human and mouse liver fibrosis tissues (Figure 1). Furthermore, we noted that SLC27A5 deficiency promoted liver fibrosis (Figure 3). We thus hypothesize that SLC27A5 overexpression might protect against liver fibrosis. To test this hypothesis, specifically overexpressed SLC27A5 in the livers of WT and *Slc27a5* ^−/−^ (KO) mice through tail vein injection of adeno‐associated virus (AAV) harboring *Slc27a5* (AAV‐*Slc27a5*) or AAV‐Control (AAV‐Con) in a CCl_4_‐induced liver fibrosis model (**Figure** **7**A). Concordantly, the re‐expression of SLC27A5 in *Slc27a5* *^−/−^* mice alleviated collagen deposition, fibrosis, and liver injury (Figure 7B–F and Figure S9A–E, Supporting Information). Both serum and liver CA accumulation was reduced in *Slc27a5* *^−/−^* mice injected with AAV‐*Slc27a5* (Figure 7G,H). Notably, WT mice with SLC27A5 overexpression exhibited a mild alleviation of liver fibrosis compared with those injected with AAV‐Control (Figure 7B–E). These results suggest that the AAV‐mediated restoration of hepatic SLC27A5 protects against CCl_4_‐induced liver fibrosis in mice.

To further investigate the potential therapeutic strategies for liver fibrosis, we examined the effect of A4250, a specific ASBT inhibitor that decreases hepatic bile acid levels by inhibiting intestinal bile acid absorption,^[^ ^26^ ^]^ on CCl_4_‐induced liver fibrosis. We subjected WT and *Slc27a5* *^−/−^* mice to CCl_4_ injection and treated them with A4250 through gavage (Figure 7I). After administering the A4250, we observed an evident improvement in liver fibrosis in *Slc27a5* *^−/−^* mice (Figure S9F, Supporting Information). Furthermore, the attenuation of hepatic collagen deposition and α‐SMA expression, along with the improvement of liver function, were evident in *Slc27a5* *^−/−^* mice with A4250 administration (Figure 7J–N; Figure S9G–J, Supporting Information). Moreover, both serum and liver CA levels were markedly decreased in *Slc27a5* *^−/−^* mice following the A4250 treatment (Figure 7O,P). Overall, these findings suggest that the re‐expression of SLC27A5 or inhibition of CA accumulation ameliorates the pathogenesis of liver fibrosis, providing a potential strategy for treating this condition.

## 3 Discussion

**Figure 8:**
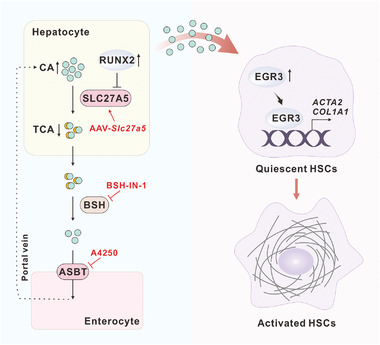
Underlying mechanisms of SLC27A5 deficiency promotes HSCs activation and fibrosis via CA. SLC27A5 was downregulated in liver fibrosis tissues via RUNX2. The loss of SLC27A5 in mice resulted in accumulating unconjugated CA in hepatocytes and subsequently activated HSCs via EGR3. Re‐expression of SLC27A5 or inhibition of CA accumulation may represent a potential strategy for liver fibrosis treatment. SLC27A5, solute carrier family 27 member 5; RUNX2, RUNX family transcription factor 2; CA, cholic acid; TCA, taurocholic acid; BSH, bile salt hydrolases; ASBT, apical sodium‐dependent bile acid transporter; EGR3, early growth response protein 3; HSC, hepatic stellate cell.

In the present study, we identified SLC27A5 as a novel regulator of liver fibrosis progression. Our findings demonstrate that the expression of SLC27A5 is diminished in the livers of cirrhotic patients and mice with liver fibrosis. Moreover, SLC27A5 deficiency aggravates the progression of liver fibrosis by activating hepatic stellate cells (HSCs) via the regulation of bile acid (BA) conjugation (**Figure** **8**). These results provide important insights into the role of unconjugated BAs in liver fibrosis.

The abnormal metabolism of BAs has been associated with various causes of liver fibrosis.^[^ ^27^, ^28^, ^29^ ^]^ However, the specific roles of BAs metabolic enzymes in liver fibrosis have not been systematically explored. In our study, we observed that the expression of the bile acid‐CoA ligase SLC27A5 was decreased in patients with cirrhosis and fibrosis mouse models. A recent study found that SLC27A5 mRNA levels were upregulated in patients with NAFLD and a NAFLD rat model;^[^ ^30^ ^]^ however, SLC27A5 expression decreased as fibrosis progressed.^[^ ^20^ ^]^ Lower expression of Slc27a5 was related to chronic CCl_4_‐induced liver fibrosis.^[^ ^31^ ^]^ Similarly, we previously suggested that SLC27A5 expression was downregulated in HCC tissues owing to DNA hypermethylation.^[^ ^32^ ^]^ This lower expression may be useful for predicting HCC prognosis.^[^ ^33^ ^]^ These findings suggest that the downregulation of SLC27A5 plays a vital role in disease progression, particularly in the development of liver fibrosis, cirrhosis, and HCC.^[^ ^34^ ^]^

The maintenance of BAs homeostasis is regulated by the farnesoid X receptor (FXR) via transcriptional repression of key enzymes involved in BAs synthesis, such as cholesterol 7α­hydroxylase (CYP7A1) and sterol 12α‐hydroxylase (CYP8B1).^[^ ^6^ ^]^ *SLC27A5* mRNA expression was upregulated after FXR agonist GW4064 treatment.^[^ ^35^ ^]^ However, the underlying mechanisms of SLC27A5 downregulation in liver fibrosis remain unclear. In this study, we identified RUNX2 as a transcriptional repressor of SLC27A5. The RUNX2 is a master transcription factor in regulating osteoblast differentiation, angiogenesis, cancer metastasis, and, in particular, the fibrosis response.^[^ ^36^, ^37^, ^38^ ^]^ The upregulation of RUNX2 promotes aortic and pulmonary fibrosis.^[^ ^39^, ^40^ ^]^ Several fibrosis‐related genes, such as *COL1A1* and *TIMP1*, are regulated by RUNX2.^[^ ^41^, ^42^ ^]^ Notably, putative RUNX2‐binding sites are localized in the *SLC27A5* promoter, and SLC27A5 expression is repressed by RUNX2, which offers at least one underlying reason for SLC27A5 downregulation in patients with cirrhosis and mice with liver fibrosis. These findings suggest SLC27A5 as a potential diagnostic marker of liver fibrosis.

Another important finding of this study was the physiological role of SLC27A5 in liver fibrosis. This finding is supported by several lines of evidence. The experimental *Slc27a5* *^−/−^* mice exhibited significantly increased collagen deposition and α‐SMA expression, with an abundance of unconjugated BAs in the serum and liver. This is consistent with a previous report of a child with a homozygous mutation in *SLC2A75* who developed extensive fibrosis.^[^ ^18^ ^]^ Although SLC27A5 expression was not reduced in the liver biopsy, over 85% of the unconjugated BAs were present in the plasma of this child, leading us to speculate that the deficiency of BAs conjugation may result in liver fibrosis.

Patients with disrupted BAs conjugation exhibited fat‐soluble vitamin deficiency, whereas some developed liver disease.^[^ ^19^ ^]^ Meanwhile, increased levels of DCA, an unconjugated bile acid from the gut microbiota, promote liver cancer through the senescence of HSCs.^[^ ^43^ ^]^ Elevated BAs levels induce cholestatic disease and fibrosis, which may be attributed to hepatocyte injury or cholangiopathy.^[^ ^10^, ^44^ ^]^ However, the role of BAs in HSC activation has not been fully explored.

TCA is significantly increased in patients with cirrhosis and promotes HSC activation.^[^ ^45^, ^46^ ^]^ Increased levels of conjugated BAs such as TDCA and glycodeoxycholate (GDCA) in patients with NASH and fibrosis mouse models could also increase the protein expression of fibrosis‐related markers in LX‐2 cells.^[^ ^47^ ^]^ However, elevated serum BAs of normal composition may not lead to liver injury or fibrosis, which is supported by the fact that patients with Na^+^‐taurocholate co‐transporting polypeptide (NTCP) deficiency present with significantly elevated levels of conjugated BAs in the plasma without distinct clinical symptoms.^[^ ^48^ ^]^ In this study, we demonstrated that SLC27A5 knockout in mice substantially increased hepatic and serum‐unconjugated CA levels; this subsequently promoted HSC activation and liver fibrosis. Meanwhile, male C57BL/6J mice fed a chow diet supplemented with 0.5% (w/w) CA reportedly exhibit significant liver fibrosis.^[^ ^49^ ^]^ Although unconjugated DCA was also increased in *Slc27a5* *^−/−^* mice, the DCA may not promote HSCs activation, which is consistent with the unchanged levels of DCA in cirrhotic patients as previously reported.^[^ ^45^ ^]^

Furthermore, attenuation of BA levels alleviated CCI_4_‐induced liver fibrosis in *Slc27a5* *^−/−^* mice. Bile acids are synthesized in the liver and secreted into the intestine to facilitate the absorption of lipophilic nutrients, with 95% of BAs being reabsorbed via the ASBT transporter.^[^ ^5^ ^]^ Thus, the interruption of ASBT has been suggested as a promising strategy to attenuate bile acid‐related diseases.^[^ ^50^ ^]^ Recently, the novel ASBT inhibitor A4250 has demonstrated promise in patients with progressive familial intrahepatic cholestasis.^[^ ^51^, ^52^ ^]^ This study suggests that A4250 reduced CA levels and liver fibrosis progression in CCI_4_‐treated *Slc27a5* *^−/−^* mice. Although A4250 did not specifically reduce CA levels in mice, the presence of abundant CA in the liver and serum of *Slc27a5* *^−/−^* mice suggests that it can be an effective therapy. Hence, we conclude that SLC27A5 deficiency promotes liver fibrosis progression by upregulating unconjugated CA levels.

Various BAs can induce the proliferation of activated rat HSCs via the epidermal growth factor receptor (EGFR) pathway. Notably, these bile acids did not affect the expression of collagen I.^[^ ^53^ ^]^ Furthermore, BAs stimulate the expression of early growth response (EGR) genes and protein kinase C signal transduction pathways in HSCs.^[^ ^54^ ^]^ However, the precise details and functions of this signaling mechanism are yet to be determined. The EGR family of transcription factors comprises four members that play a role in immune regulation.^[^ ^55^ ^]^ Notably, a specific member of this family, EGR3, reportedly modulates TGF‐β1 transcription in T cells.^[^ ^56^ ^]^ This member also exerts a profibrotic role in skin and cardiac fibroblasts by stimulating the expression of *ACTA2* and *COL1A1* genes.^[^ ^25^, ^57^ ^]^ Our RNA‐seq data revealed a significant upregulation of EGR3 in CA‐treated LX‐2 cells. To further determine the specific role of EGR3, we conducted an experiment to knock down EGR3 using shRNA. The results suggested that the knockdown of EGR3 attenuated the activation of HSCs induced by CA treatment, indicating the importance of unconjugated CA in HSC activation and fibrosis. However, the specific mechanism underlying the upregulation of EGR3 in CA‐treated HSCs requires further investigation.

In the present study, we have mainly focused on the role of SLC27A5 in regulation of BAs conjugation during the progression of liver fibrosis. However, other functions of SLC27A5, such as transportation of LCFA ^[^ ^14^ ^]^ in liver fibrosis, need further investigation. Additionally, the injection of AAV‐*Slc27a5* at an earlier time point may be a better choice to investigate the effects of SLC27A5 overexpression on the treatment of liver fibrosis.

In summary, these results suggest that SLC27A5 deficiency in mice promotes liver fibrosis by inhibiting BAs conjugation. This leads to the accumulation of unconjugated CA in the liver, which activates HSCs via EGR3. These findings provide novel insights into the role of SLC27A5 in the progression of fibrosis. The dysregulation of BAs composition is thus suggested to be closely linked to HSC activation. Therefore, the restoration of SLC27A5 expression in hepatocytes and repression of profibrotic CA levels represent potential applications in the treatment of fibrosis.

## 4 Experimental Section

### Clinical Samples

Liver tissue samples from healthy controls (n = 14) and cirrhotic patients (n = 20) were obtained from the Second Affiliated Hospital of Chongqing Medical University. Fasting serum samples were collected from healthy controls (n = 18) and cirrhotic patients (n = 18) recruited from the Second Affiliated Hospital of Chongqing Medical University. Fasting blood specimens were collected from all volunteers and centrifuged at 2000 g for 10 min at 4 °C. The resulting sera were collected and stored at −80 °C until analysis. Clinical characteristics of participants were summarized in Table S1 (Supporting Information). Informed consents were obtained from all involved participants. This study was approved by the Research Ethics Committee of Chongqing Medical University (Approval number: 2022054).

### Cell Cultures and Reagents

Human hepatic stellate cell line LX‐2 was obtained from the China Center for Type Culture Collection (CCTC, Wuhan, China). An immortalized human liver cell line MIHA was kindly provided by Prof. Ben C. B. Ko (The Hong Kong Polytechnic University, Hong Kong, China).^[^ ^58^ ^]^ Cells were cultured in Dulbecco's modified Eagle's medium (DMEM; Gibco, Grand Island, NY, USA) supplemented with 10% FBS (Corning, NY, USA), and 1% penicillin‐streptomycin (MedChemExpress, NJ, USA) at 37 °C containing 5% CO_2_.

For the bile acid treatment experiments, LX‐2 cells were seeded in 6‐well plates, and cells were stimulated by fresh 2% FBS medium with or without individual BAs (Sigma‐Aldrich, St Louis, MO, USA) at 25, 50, 75, and 100 µм for 48 h, and DMSO were used as a vehicle control.

For co‐culture experiments, LX‐2 cells were seeded in 12‐well plates at densities of 50000 cells cm^−2^. MIHA cells were seeded and adhered to the porous polyester (PET) membrane surface of trans‐well inserts (0.4 µm pores, BIOFIL, Guangzhou, China) at a density of 100000 cells cm^−2^. Inserts containing MIHA cells were incubated overnight to adapt to the new conditions before being placed in 12‐well plates containing HSCs. The co‐culture system was incubated for 48 h in DMEM medium with 2% FBS. Trans‐well inserts were then carefully removed and the LX‐2 cells in 12‐well plates were immediately collected for analysis.

### Animals

Heterozygous C57BL/6N‐Slc27a5^em1cyagen^ mice were created using the CRISPR‐Cas9 technology by Cyagen Biosciences (Suzhou, China) and were crossed to breed wild‐type (WT) and *Slc27a5* ^−/−^ mice. The genotype of WT and *Slc27a5* ^−/−^ mice was confirmed by PCR amplification of tail DNA (PCR primers were listed in Table S2, Supporting Information). All mice were maintained under specific pathogen‐free conditions in the laboratory animal center of Chongqing Medical University. All animal experiments were performed under the guidelines of the institutional Animal Care and Use Committee at Chongqing Medical University. All animal procedures were also approved by the Animal Experimentation Ethics Committees of Chongqing Medical University (Approval number: 2022054).

For the carbon tetrachloride (CCl_4_) model of liver fibrosis, 6‐ to 8‐week‐old male WT and *Slc27a5* ^−/−^ mice were given intraperitoneal (i.p.) injections of CCl_4_ (1.0 mL kg^−1^ body weight, dissolved in corn oil at a ratio of 1:9) (Macklin, Shanghai, China) or vehicle (corn oil) twice a week for 6 weeks (n = 5 per group). For the thioacetamide (TAA) model of liver fibrosis, 6‐ to 8‐week‐old male WT and *Slc27a5* ^−/−^ mice were intraperitoneally injected with phosphate‐buffered saline (PBS) or TAA (100 mg kg^−1^ body weight) (Macklin, Shanghai, China) three times a week for 8 weeks (n = 5 per group). The mice were starved overnight and sacrificed 2 days after the final injection. The mouse livers and serum were collected for subsequent experiments. Mouse serum ALT, AST, ALP. and TBil were detected using an automatic biochemical analyzer (Catalyst One, IDEXX, USA).

For overexpression of SLC27A5, AAV8‐TBG‐control or AAV8‐TBG‐*Slc27a5* (OBiO Technology, Corp., Ltd. Shanghai, China) was injected via tail vein of WT and *Slc27a5* ^−/−^ male mice at 8 weeks of age (2 × 10^11^ genome copies per mouse). After 8 weeks of CCI_4_ injection, mice were starved overnight and sacrificed for analysis.

For inhibition of the hepatic CA accumulation, 8‐week‐old male WT and *Slc27a5* *^−/−^* mice were subjected to the CCl_4_ injection for 6 weeks and treated with vehicle or bile acid transporter inhibitor A4250 (10 mg kg^−1^ body weight) (HY‐109120, MedChemExpress) by daily gavages in the last 4 weeks. After 4 weeks of A4250 treatment, mice were starved overnight and sacrificed for analysis.

For treatment of BSH‐IN‐1, Adult male WT or *Slc27a5* *^−/−^* mice were gavaged with a single dose of BSH‐IN‐1 (10 mg kg^−1^, TargetMol, USA) or vehicle control (n = 3 per group). The primary mice hepatocytes were isolated after 24 h of the gavage to perform the co‐culture experiment.

### Extraction and Profiling of Bile Acids

Serum bile acids extraction was performed by mixing 50 µL of serum with 500 µL of methanol containing 20 µL of 1 µg mL^−1^ of Cholic acid‐d4 (CDN Isotopes Inc, Pointe‐Claire, Canada) used as the internal standard. For liver bile acid extraction, ≈60 mg of frozen liver samples was weighed and homogenized in 600 mL of methanol. An amount of 500 µL of liver homogenate was spiked with 20 µL of internal standards. The serum or liver mixture was then vortexed at 12000 g at 4 °C for 30 seconds and incubated for 30 min at 65 °C. The samples were then heated on boiling water bath for 3 min and cooled to room temperature. The mixture was centrifuged at 12,000 g for 10 min at 4 °C, and the supernatant was collected. The pellet was resuspended in 500 µL of methanol, vortexed for 2 min, and centrifuged at 12,000 g for 10 min at 4 °C. The supernatant was combined with that collected earlier, and dried under vacuum. The residue was redissolved with 50% methanol to a final volume of 100 µL. After centrifugation at 12000 g at for 10 min at 4 °C, an aliquot of 70 µL of supernatant was used for liquid chromatography‐tandem mass spectrometry (LC‐MS) analysis as previously described.^[^ ^59^ ^]^ Briefly, the separation and detection were performed on a Waters ACQUITY UPLC CSH C18 column (2.1 ×100 mm, 1.7 µm) with an Agilent 1290—6495 C ultra performance liquid chromatography‐triple quadrupole tandem mass spectrometry system. Ammonium formate solution of 5 mм with 0.1% formic acid was used as mobile phase A, and methanol was chosen as mobile phase B. The dynamic multiple reaction monitoring (DMRM) was used for MS analysis.

For bile acid quantitation, calibration curves of standard samples containing various bile acids were used according to recently published methods.^[^ ^59^ ^]^ Serial standard mixtures were obtained by gradient dilution (4 ×). The standard mixtures were then mixed with internal standard and underwent the same sample preparation as the test samples. Bile acid quantitation was based on the peak area ratio of the targeted bile acids to Cholic acid‐d4. The bile acids concentrations of test samples were quantified using slopes, but not intercepts, of the calibration curves to deduct background signals.

### Primary Mouse Liver Cell Isolation and Culture

Primary murine hepatocytes were isolated from the livers of male WT and *Slc27a5* ^−/−^ mice aged 8–12 weeks according to a reported protocol ^[^ ^60^ ^]^ with some modification that includes the following steps: in situ pronase (P5147, Sigma‐Aldrich) /collagenase (V900893, Sigma‐Aldrich) perfusion of mouse liver, perfused livers were minced, filtered through 70 µm cell strainer (BS‐70‐XBS, Biosharp, Beijing, China), and centrifuged at 40 g for 5 min at 4 °C to separate hepatocytes. Hepatocytes were plated in Dulbecco's modified Eagle's medium with 1 g L^−1^ glucose (DMEM‐low glucose, SH30021.01, HyClone) supplemented with 5% FBS, 15 mmol L^−1^ HEPES (H1095, Solarbio, Beijing, China), and 1% penicillin‐streptomycin. Cells were maintained overnight in serum‐free DMEM containing 1 g L^−1^ glucose. After 48 h of cell seeding in coated plates, medium was collected and concentrated by a speed‐vacuum to measure bile acids by LC‐MS.

HSCs were isolated according to a previously published method,^[^ ^61^ ^]^ and the supernatant was further centrifuged at 580 g for 10 min at 4 °C, resuspended in density gradient‐based Nycodenz (1002424‐1, Alere Technologios AS), and centrifuged at 1400 g for 17 min at 4 °C. HSCs were collected from the interface and cultured in DMEM with 10% FBS and 1% penicillin‐streptomycin.

The 48 h culture supernatant from primary hepatocytes of WT or *Slc27a5* *^−/−^* mice was collected and centrifuged at 2000 g for 5 min to prepare it for the conditional culture. The supernatant was collected and labelled as conditioned medium. Twenty‐four hours post isolation, the medium of the primary HSCs was replaced by the conditioned medium from WT or *Slc27a5* *^−/−^* mice primary hepatocytes and incubated for another 48 h. For TGFβ1 treatment, the primary HSCs after 24 h isolation were treated with and without TGFβ1 (4 ng mL^−1^, Novoprotein, Beijing, China) for another 48 h. For CA treatment, the primary HSCs after 24 h isolation were treated with CA (50 µм) for another 48 h.

### Western Blotting

Proteins were extracted from cells or liver tissues in the lysis buffer (Beyotime, Shanghai, China) consisting of protease inhibitor cocktail (1:100, TargetMol, MA, USA). The concentration of proteins was determined using BCA Protein Assay kit (Beyotime). The extracted proteins were separated by 10% SDS/PAGE and electro‐transferred to PVDF membranes (Millipore, Billerica, MA, USA). After blocking in 5% milk for 1 h, the membranes were probed with primary antibodies against SLC27A5 (1:1000, NBP2‐37412, Novus Biologicals, CO, USA), α‐SMA (1:1000, ER1003, Huabio, Hangzhou, China), COL1A1 (1:1000, WL0088, Wanleibio, Shenyang, China), COL3A1 (1:1000, 22734‐1‐AP, Proteintech, IL, USA), RUNX2 (1:1000, 20700‐1‐AP, Proteintech, IL, USA), EGR3 (1:500, sc‐390967, Santa Cruz Biotechnology, USA), β‐actin (1:3000, BL005B, Biosharp), or GAPDH (1:3000, AG019‐1, Beyotime, Shanghai, China) at 4 °C overnight. Membranes were then incubated with horseradish peroxidase‐conjugated secondary antibody (Abcam, Cambridge, UK). The staining was visualized using ClarityTM Western ECL Substrate (Bio‐Rad, CA, USA).

### Liver histological and Immunohistological (IHC) Staining

Liver specimens were fixed in 4% paraformaldehyde, embedded in paraffin and cut into 4 µm sections. Then, the specimens were deparaffinized, hydrated and stained by standard methods. To examine hepatic morphology and assess liver fibrosis, H&E and Sirius Red staining were performed. Sections were immune‐stained for SLC27A5 (1:500, NBP2‐37412, Novus Biologicals, CO, USA), α‐SMA (1:300, 19245T, CST, MA, USA), F4/80 (1:300, 70076T, CST), or RUNX2 (1:300, 20700‐1‐AP, Proteintech, IL, USA) overnight at 4 °C. Sections were then incubated with a secondary anti‐rabbit or anti‐mouse IgG (ZSGB‐BIO, Beijing, China) and stained using 3,3′‐diaminobenzidine (ZSGB‐BIO). Stained slides were scanned with a Pannoramic Scan 250 Flash and images were acquired using Pannoramic Viewer 1.15.2 (3DHistech, Budapest, Hungary). Immunostaining and Sirius Red staining were quantified by threshold analysis using the NIH ImageJ software. The immunohistochemical staining of SLC27A5 and RUNX2 was semi‐quantitatively analyzed using the immunoreactive scoring system.^[^ ^62^ ^]^

### Hepatic Hydroxyproline (HYP) Measurement

The HYP levels of fresh liver samples of mice were quantified. Concentration was calculated by a standard curve using the HYP Content Assay Kit (BC0255, Solarbio, China) according to the manufacturer protocol.

### Immunofluorescence

HSCs were seeded and treated with CA (50 µм) in slide chambers. After being fixed in 4% paraformaldehyde, cells were permeabilized in 0.5% Triton X‐100 for 10 min. After being blocked in 5% goat serum for 1 h, slides were incubated with primary antibody against α‐SMA (1:300, 19245T, CST) overnight at 4 °C. The slides were then washed 5 times in PBS and incubated with goat anti‐rabbit conjugated with Alexa Fluor 488 or 552 secondary antibody (1:100, ZF‐0311, ZSGB‐bio) for 1 h at 37 °C. Nucleus was stained using 1 µg mL^−1^ 4′,6‐diamidino‐2‐phenylindole (DAPI,1:2000, Roche, Basel, Switzerland). Stained sections were observed by a laser‐scanning confocal microscope (Leica TCS SP8, Solms, Germany).

### Quantitative Reverse Transcriptase PCR (qRT‐PCR)

Total RNA of tissue or cells was isolated with Trizol reagent (Invitrogen, Rockville, MD). Reverse transcription (RT) reactions were performed using PrimeScript RT Reagent Kit (RR047A, TaKaRa, TKY, Japan). Real‐time PCR was carried out using Bio‐Rad CFX96 machine (Bio‐Rad, Hercules, CA, USA). The level of GAPDH or β‐actin RNA expression was used to normalize the data. The sequences of qRT‐PCR primers were listed in Table S2 (Supporting Information).

### Construction of Plasmids

The expression vector of human *RUNX2* was inserted into the *Sal* I and *Xba* I sites of the shuttle vector pAdTrack‐TO4 (from Dr T‐C He, University of Chicago, USA). The 5′‐flanking region (from −2023 to +366 nt) of *SLC27A5* gene was inserted into the *Hind* III and *Xho* I sites of the pGL3‐Basic vector (Promega, Madison, WI, USA), named pGL3‐P1. To construct another length of luciferase reporter plasmid of SLC27A5, the region from −1001 to +182 nt of *SLC27A5* gene was inserted into the pGL3‐Basic vector, named pGL3‐P2. Oligonucleotide sequences were listed in Table S2 (Supporting Information).

### Lentivirus‐Meditated RNA Interference

The small double‐strand hairpin shRNA expressing constructs (sh*RUNX2*, sh*EGR3*, and sh*Egr3*) were designed and annealed into the *Hpa* I and *Xho* I sites of pLL3.7 lentivirus vector (kindly provided by Prof. Bing Sun, Center for Excellence in Molecular Cell Science, Chinese Academy of Sciences, Shanghai, China). A negative control construct (shCon) was also generated. The lentiviral supernatants were produced in HEK293T cells as previously described.^[^ ^63^ ^]^ Oligonucleotide sequences were listed in Table S2 (Supporting Information).

### CRISPR/Cas9‐Mediated Knockout Cells

SLC27A5‐knockout cells were constructed by the CRISPR‐Cas9 system provided by Prof. Ding Xue (Tsinghua University, Beijing, China), as previously described.^[^ ^32^ ^]^ The Knockout of SLC27A5 in MIHA cells were validated by immunoblotting. The sgRNA target sequences were listed in Table S2 (Supporting Information).

### Luciferase Reporter Assay

MIHA cells were transfected in twelve‐well plates containing 4 µL of Lipofectamine 8000, 0.5 µg of pADTrack‐TO4 control or pADTrack‐RUNX2 overexpression vectors, 0.5 µg of each luciferase reporter plasmids pGL3‐basic (Promega, Madison, WI, USA), pGL3‐P1 (from −2023 to +366 nt of the promoter region of the *SLC27A5* gene), or pGL3‐P2 (from −1001 to +182 nt of the promoter region of the *SLC27A5* gene), and 10 ng of pRL‐TK‐Renilla (as transfection control) for 48 h. Cells were harvested and assayed for luciferase activity using the Dual Luciferase Assay Kit (Promega, Madison, WI, USA). All experiments were performed at least three times and expressed as mean ± SEM.

### Chromatin Immunoprecipitation (ChIP) Assay

MIHA cells of 6 × 10^6^ were cross‐linked using 1% paraformaldehyde for 8 min at 37 °C. Cell lysates were sonicated at 30% power for 10 cycles (15 seconds ON and 15 seconds OFF). Supernatants were separated and incubated with anti‐RUNX2 (20700‐1‐AP, Proteintech, IL, USA), anti‐EGR3 (sc‐390967, Santa Cruz Biotechnology, USA), or control IgG overnight at 4 °C. Chromatin‐antibody complexes were collected by protein A/G agarose beads (Millipore), washed and then eluted. Real‐time PCR was used to analyze the RUNX2‐binding DNA fragments from ChIP assays. The ChIP and ChIP‐qPCR assays were performed as previously described.^[^ ^63^ ^]^ Primers were listed in Table S2 (Supporting Information).

### RNA‐Seq Analysis

Total RNA was extracted in LX‐2 cells treated with CA (50 µм) or vehicle for 48 h using Trizol reagent and subjected to library preparation. RNA‐seq was performed by Majorbio Bio‐pharm Technology Co.,Ltd (Shanghai, China). Differential expression genes (DEGs) were analyzed using the DESeq2. Heatmap of DEGs was performed using the “ggplot2” packages in R (v4.2.1, The R Project for Statistical Computing, Vienna, Austria).

### Online Database Analysis

UCSC (https://genome.ucsc.edu/) was used to predict transcriptional factors (TFs) that could affect SLC27A5.^[^ ^64^ ^]^ JASPAR (http://jaspar.genereg.net/) was used to predict the potential binding sites of TFs on *SLC27A5* promoter.^[^ ^21^ ^]^

### Gene Expression Omnibus Database Mining

Four data sets from GEO database (https://www.ncbi.nlm.nih.gov/geo/) were analyzed with GEO2R to profile gene expression between different samples, such as mild fibrosis versus advanced fibrosis in NAFLD patients (GSE31803), control versus NASH patients (GSE48452), normal versus cirrhosis patients (GSE25097), and different stage of fibrosis in HBV infected patients (GSE84044).

### Statistical Analysis

All data were presented as the means ± SEM. Tests used to examine the differences between groups include Student's *t* test, one‐way ANOVA and with the Tukey's post hoc test, and two‐way ANOVA with Tukey's multiple comparisons test. Pearson correlation coefficient (r) was used to test the linear correlation. *P*‐values < 0.05 were considered statistically significant. *P < 0.05, **P < 0.01, ***P < 0.001. Statistical analyses were conducted using GraphPad Prism 8.0 software (La Jolla, CA, USA).

### Ethics Approval Statement

This study was approved by the Research Ethics Committee of Chongqing Medical University (approval number: 2022054). All animal experiments were performed under the guidelines of the institutional Animal Care and Use Committee at Chongqing Medical University.

### Patient Consent Statement

Informed consents were obtained from all involved participants.

## Conflict of Interest

The authors declare no conflict of interest.

## Author Contributions

K.W., Y.L., J.X., and J.L. contributed equally to this work. N.T., C.C. and A.H. conceived and designed the study. K.W., Y.L., J.X., and J.L. performed most experiments and analyzed the data. K.W. provided suggestions and designed primer sequence. H.L. constructed plasmids. F.X., D.L., D.N., and X.T. assisted with mice experiments. C.C. provided technical assistance. K.W. and N.T. wrote the manuscript with all authors providing feedback. The order of the co‐first authors was determined on the basis of their relative contributions to the study.

## Supporting information

Supporting Information

Supporting Information

## Data Availability

The data that support the findings of this study are available from the corresponding author upon reasonable request.
