# Supplementary material for: Loss of SLC27A5 Activates Hepatic Stellate Cells and Promotes Liver Fibrosis via Unconjugated Cholic Acid
Source: Adv Sci (Weinh). 2023 Nov 13;11(2):2304408. doi: 10.1002/advs.202304408 (PMC10787101; doi:10.1002/advs.202304408)
Supplement: Supplementary file 1 — Supporting Information [file ADVS-11-2304408-s001.pdf]

ADVANCED SCIENCE
Open Access

Supporting Information

for *Adv. Sci.*, DOI 10.1002/advs.202304408

Loss of SLC27A5 Activates Hepatic Stellate Cells and Promotes Liver Fibrosis via Unconjugated Cholic Acid

Kang Wu, Yi Liu, Jie Xia, Jiale Liu, Kai Wang, Huijun Liang, Fengli Xu, Dina Liu, Dan Nie, Xin Tang, Ailong Huang, Chang Chen* and Ni Tang**

Supporting Information

Loss of SLC27A5 Activates Hepatic Stellate Cells and Promotes Liver Fibrosis via Unconjugated Cholic Acid

Kang Wu, Yi Liu, Jie Xia, Jiale Liu, Kai Wang, Huijun Liang, Fengli Xu, Dina Liu, Dan Nie, Xin Tang, Ailong Huang, Chang Chen*, Ni Tang**

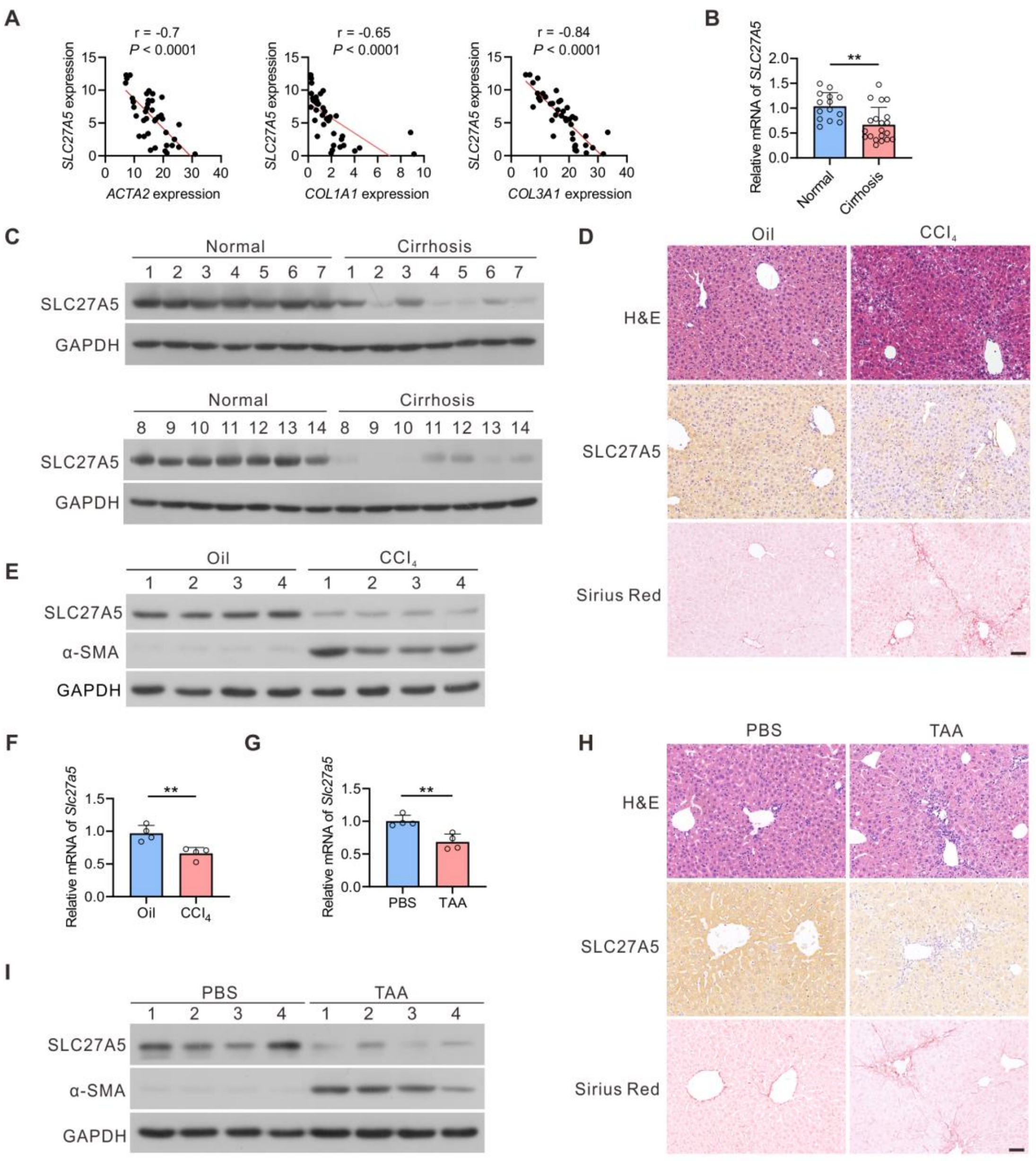

Figure S1. SLC27A5 expression is generally down-regulated in human and murine fibrotic livers.

(A) Correlation of hepatic *SLC27A5* mRNA with fibrosis-related genes (*ACTA2*, *COL1A1* and *COL3A1*) in cirrhotic patients (n = 40 for GSE25097). (B) Transcript levels of *SLC27A5* in normal and cirrhosis livers (n=14 or 20 each). (C) Representative bands of SLC27A5 in healthy and fibrotic livers (n=14 per group) were assessed by Western blotting assay. (D-F) WT mice were treated with oil or CCl_4 for six weeks (n = 4 per group). (G-I) WT mice were treated with PBS or TAA for

eight weeks (n = 4 per group). (D, H) Representative liver histology of H&E, Sirius Red staining and IHC staining for SLC27A5. Scale bar, 50 μm. (E, I) Expression of SLC27A5 and α-SMA was determined by western blotting. (F, G) Hepatic mRNA levels of *Slc27a5* were measured by qRT-PCR. Data are mean ± SEM. $^{**}P < 0.01$. Data in (A) were analyzed by Pearson correlation coefficient analysis. Data in (B) and (F-G) were analyzed by two-tailed Student's *t* test.

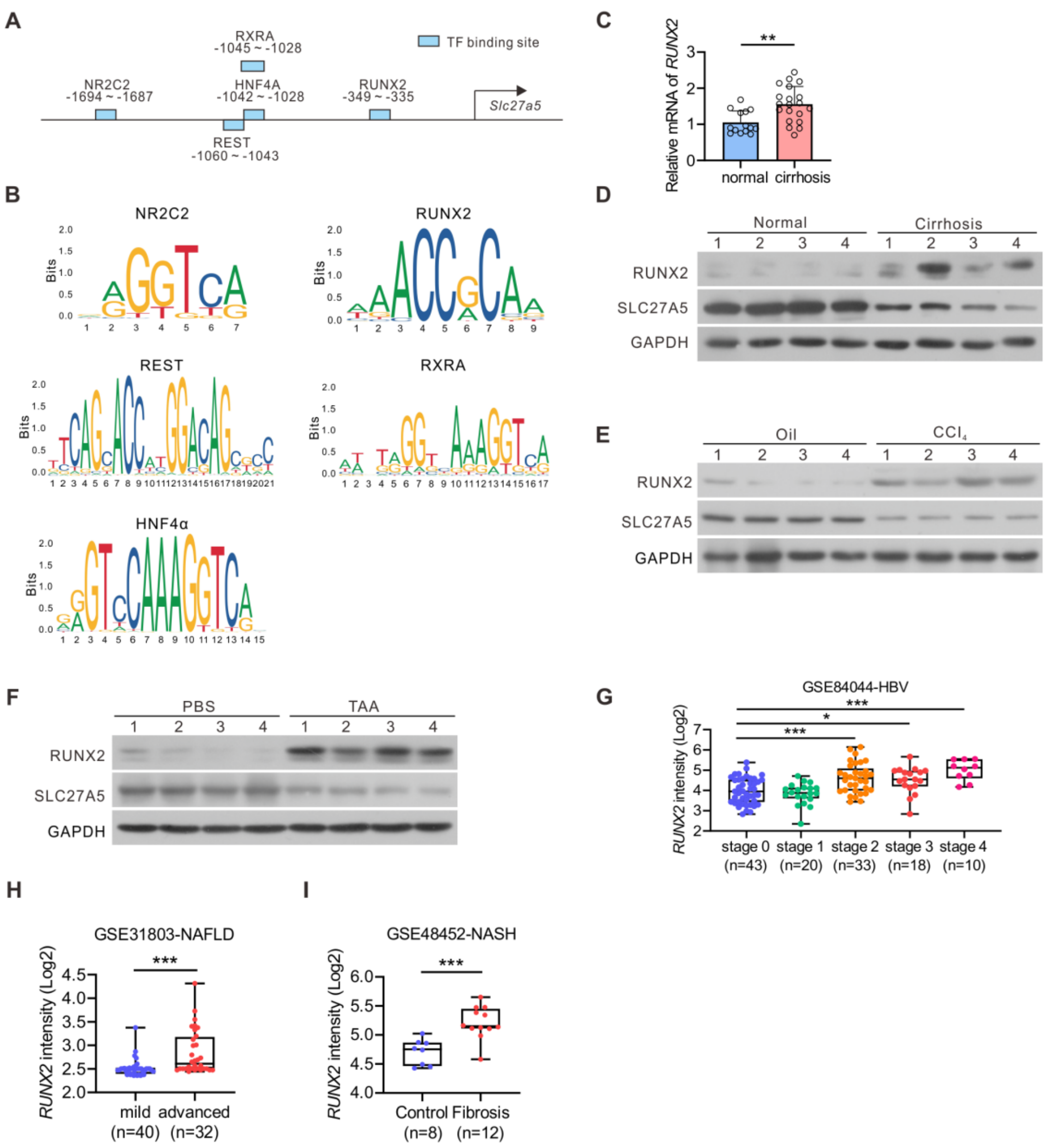

Figure S2. Elevated expression of RUNX2 downregulates SLC27A5 in fibrosis liver.

(A) The predicted transcription factors and its binding site on SLC27A5 gene promoter region by using JASPAR. (B) Putative motifs for NR2C2, REST, RXRA, HNF4α and RUNX2 found in the *SLC27A5* promoter. (C) RT-qPCR analysis of *RUNX2* expression in normal and cirrhosis livers (n=14 or 20 each). (D) Representative bands of RUNX2 and SLC27A5 in normal and cirrhosis livers (n=4 for per group) were analyzed by Western blotting assay. (E-F) Hepatic protein

expression of RUNX2 and SLC27A5 in WT mice subjected to the CCl_4 (E) and TAA (F) model (n = 4 per group). (G-I) Box plots of relative mRNA levels of *RUNX2* in GSE84044 (G), GSE31803 (H), and GSE48452 (I) dataset. Data are mean ± SEM. *$P < 0.05$, **$P < 0.01$, ***$P < 0.001$. Data in (C) and (H-I) were analyzed by two-tailed Student's *t* test. Data in (G) and were analyzed by one-way ANOVA with the Tukey's post hoc test.

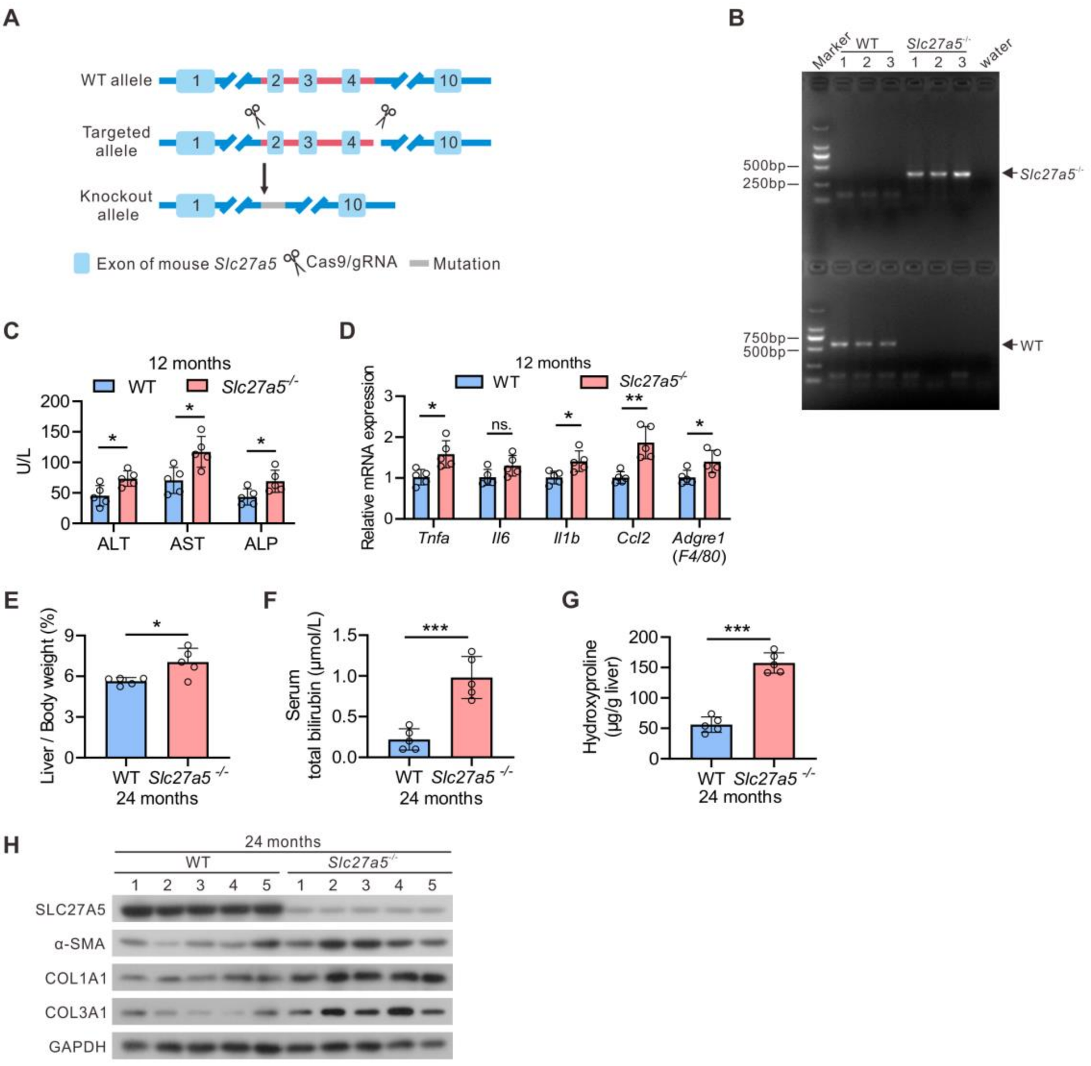

Figure S3. SLC27A5 deficiency in mice induces spontaneous hepatic fibrosis.

(A) Schematic representation of the creation of *Slc27a5*$^{-/-}$ mice by CRISPR-Cas9 gene targeting. (B) The identification of *Slc27a5*$^{-/-}$ genotype by PCR amplification. (C) Serum ALT, AST, and ALP levels from 12-month-old WT and *Slc27a5*$^{-/-}$ mice (n = 5 per group). (D) Relative mRNA levels of inflammatory genes of liver tissues from 12-month-old WT and *Slc27a5*$^{-/-}$ mice (n = 5 per group). (E) Hepatomegaly in 24-month-old WT and *Slc27a5*$^{-/-}$ mice represented by the ratio of liver to body weight (n = 5 per group). (F) Serum total bilirubin levels from 24-month-old WT and *Slc27a5*$^{-/-}$ mice (n = 5 per group). (G) Hepatic hydroxyproline content was measured in 24-month-old WT and *Slc27a5*$^{-/-}$ mice (n = 5 per group). (H) The hepatic protein

levels of α-SMA, COL1A1 and COL3A1 in liver tissues from 24-month-old WT and *Slc27a5*$^{-/-}$ mice (n = 5 per group). Data are mean ± SEM. **P* < 0.05, ****P* < 0.001, two-tailed Student's *t* test.

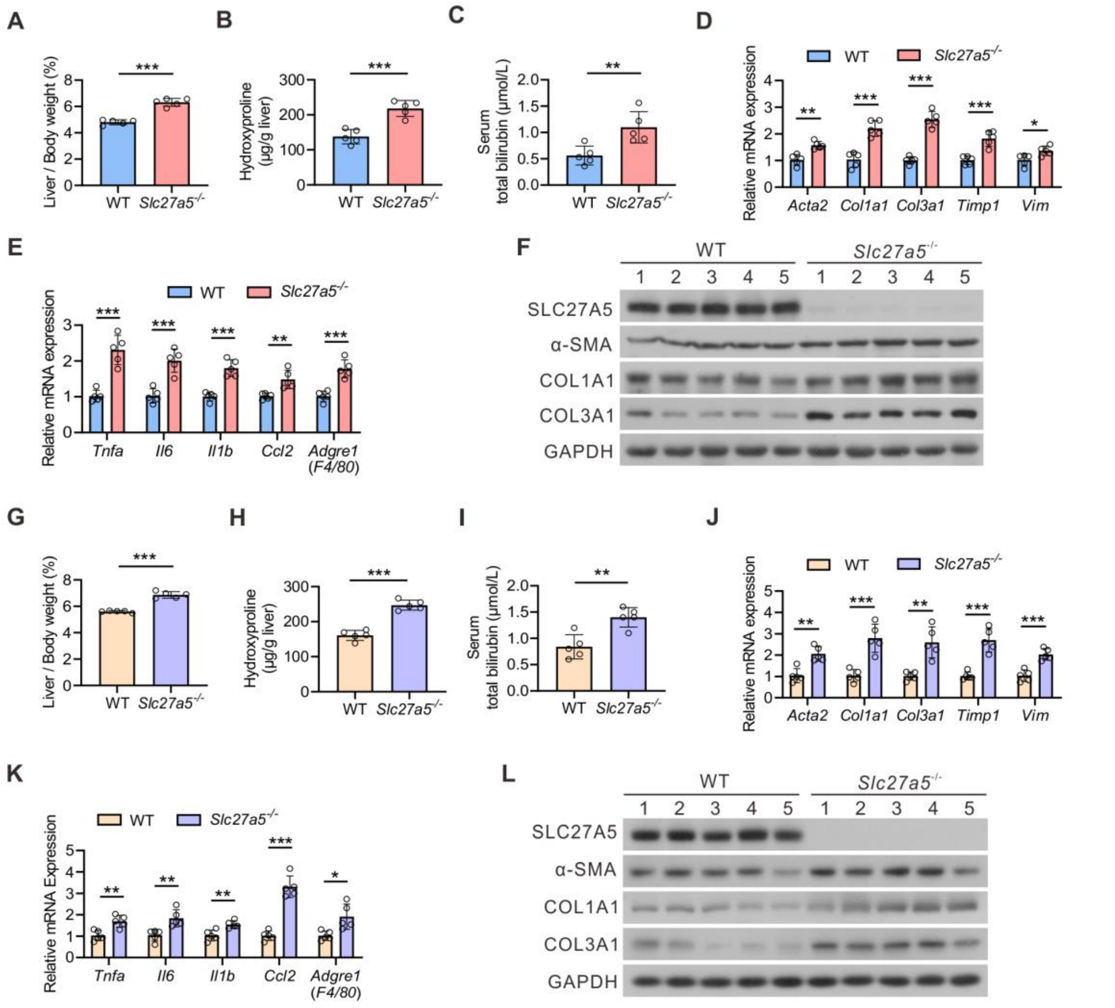

Figure S4. SLC27A5 deficiency promotes CCl_4- and TAA-induced liver fibrosis. (A-F) Eight-week-old male WT and *Slc27a5*$^{-/-}$ mice were injected with CCl_4 for six weeks (n = 5 per group). (G-L) Eight-week-old male WT and Slc27a5$^{-/-}$ mice were injected with TAA for eight weeks (n = 5 per group). (A, G) Hepatomegaly in mice represented by the ratio of liver to body weight. (B, H) Hepatic hydroxyproline content was measured. (C, I) The serum levels of total bilirubin were measured. (D-E, J-K) Hepatic mRNAs of profibrotic genes (D, J) and inflammatory genes (E, K) were measured by RT-qPCR assays. (F, L) Immunoblotting analysis of α-SMA, COL1A1, and COL3A1 expression in the mice livers. Data are mean ± SEM. *$P < 0.05$, **$P < 0.01$, ***$P < 0.001$, two-tailed Student's t test.

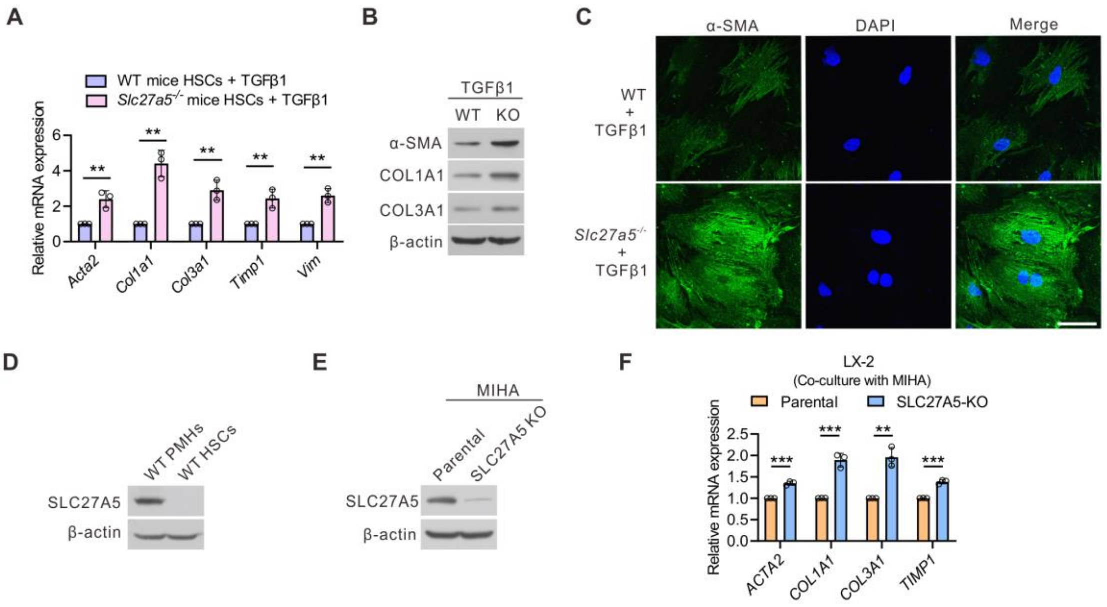

Figure S5. SLC27A5 loss in hepatocytes promotes HSCs activation in vitro.

(A-C) Primary HSCs isolated from WT and *Slc27a5*$^{-/-}$ mice were stimulated by TGFβ1 (4ng/ml) for 48 hours (n = 3 per group). mRNA expression of fibrogenic genes (A), protein expression of α-SMA, COL1A1, and COL3A1 (B), and immunofluorescence of α-SMA (C) were shown. Scale bar: 25 μm. (D) Protein expression of SLC27A5 in PMHs and HSCs of WT mice was shown. (E) Protein expression of SLC27A5 in parental and SLC27A5-KO MIHA cells was shown. (F) LX-2 cells were co-cultured with parental and SLC27A5-KO MIHA cells for 48 hours. mRNA expression of fibrogenic genes was shown. Data are mean ± SEM. **P* < 0.05, ***P* < 0.01, ****P* < 0.001, two-tailed Student's *t* test.

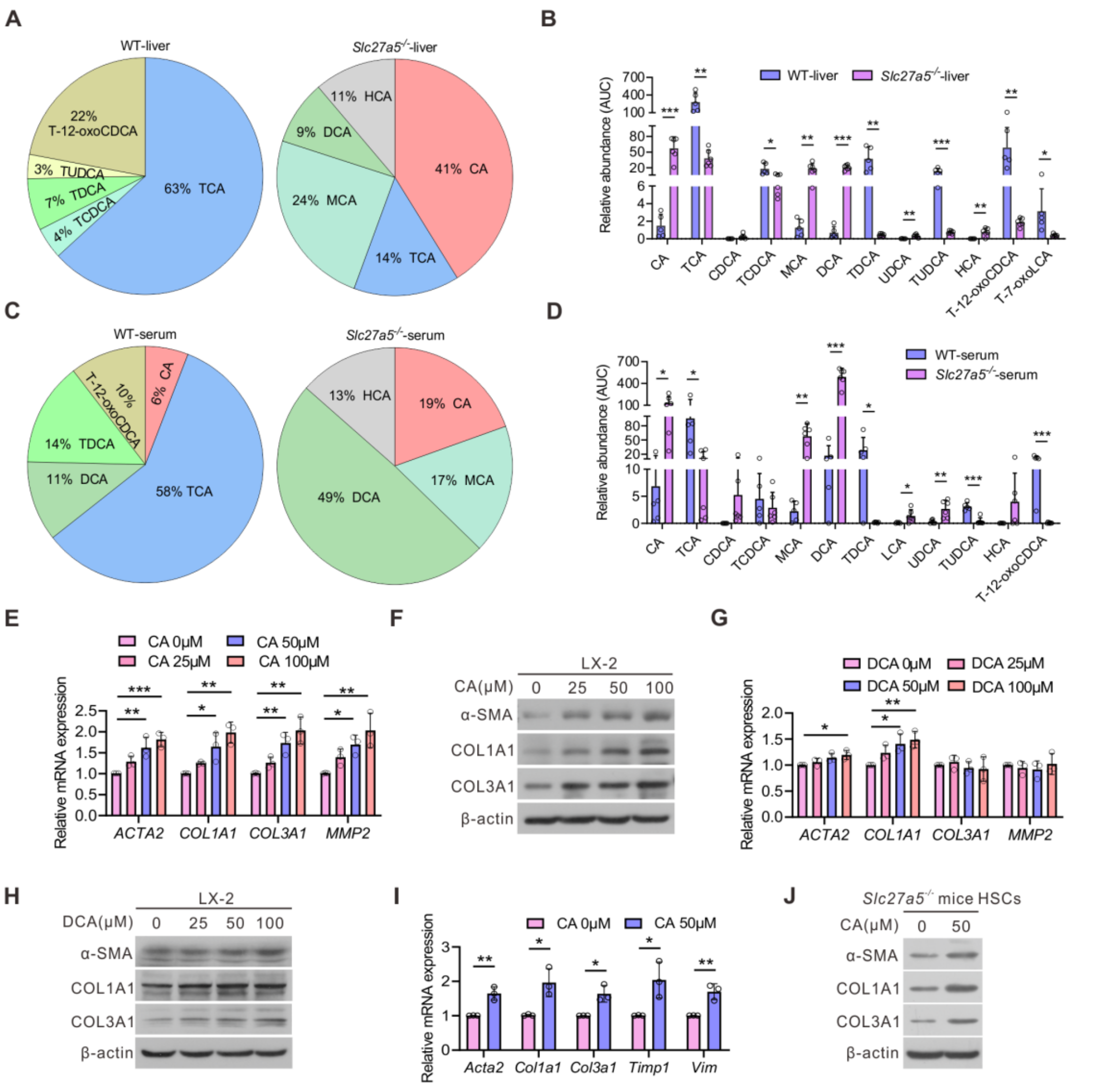

Figure S6. Unconjugated CA activates HSCs.

(A-D) Bile acid composition of serum and liver tissues in WT and *Slc27a5*$^{-/-}$ mice at 6 months of age (n=5 or 6 each). Pie graphs of the mean per cent of main bile acids (A, C), and the area under the curve (AUC) of individual bile acids peaks (B, D) were shown. (E-H) LX-2 cells were treated with CA (E-F) or DCA (G-H) at 25, 50, and 100 μM for 48hours. (E, G) mRNA expression of fibrogenic genes was measured (n=3 per group). (F, H) Protein expression of α-SMA, COL1A1, and COL3A1 was shown. (I-J) The primary HSCs isolated from adult *Slc27a5*$^{-/-}$ mice were treated with vehicle or CA at 50 μM for 48h. The mRNA expression of fibrogenic genes (I) and protein expression of α-SMA, COL1A1, and COL3A1 (J) were displayed. Data are mean ±

SEM. *$P < 0.05$, **$P < 0.01$, one-way ANOVA with the Tukey's post hoc test.

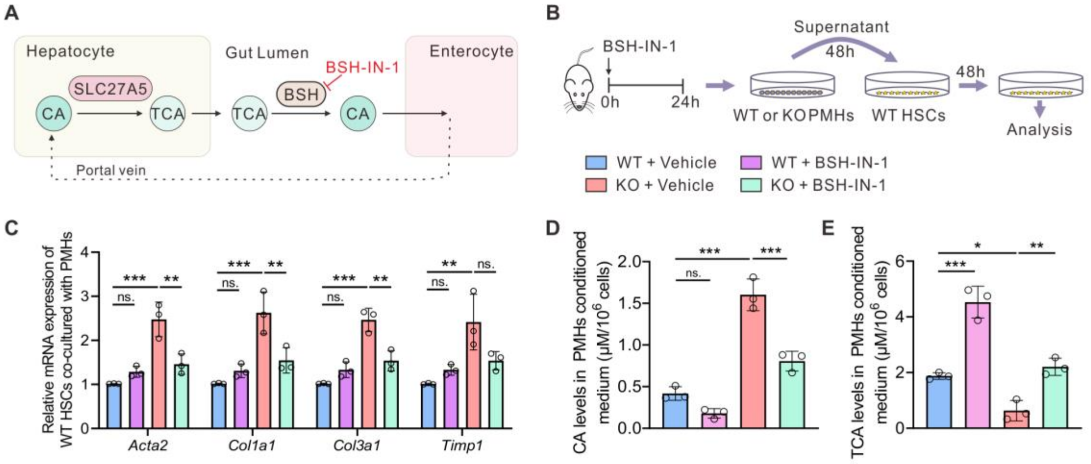

Figure S7. Inhibition of intestinal bile acid deconjugation decreases HSC activation induced by the PMH supernatant from *Slc27a5*$^{-/-}$ mice.

(A) Schematic of the target of BSH-IN-1. (B-E) Adult male WT or *Slc27a5*$^{-/-}$ (KO) mice were treated with a single dose of BSH-IN-1 (10 mg/kg) or vehicle control (n=3 per group). (B) Schematic of co-culture experiments. The PMHs were isolated after 24h of the gavage. The 48h supernatant of PMHs were collected and co-cultured with HSCs of normal WT mice for another 48 hours. (C) The mRNA expression of fibrogenic genes in HSCs co-cultured with PMHs were displayed (n=3 per group). (D) CA levels were measured in the PMHs supernatant described above (n=3 per group). (E) TCA levels were measured in the PMHs supernatant (n=3 per group). Data are presented as mean ± SEM. *$P < 0.05$, **$P < 0.01$, ***$P < 0.001$, ns., not significant, one-way ANOVA with Tukey's post hoc test.

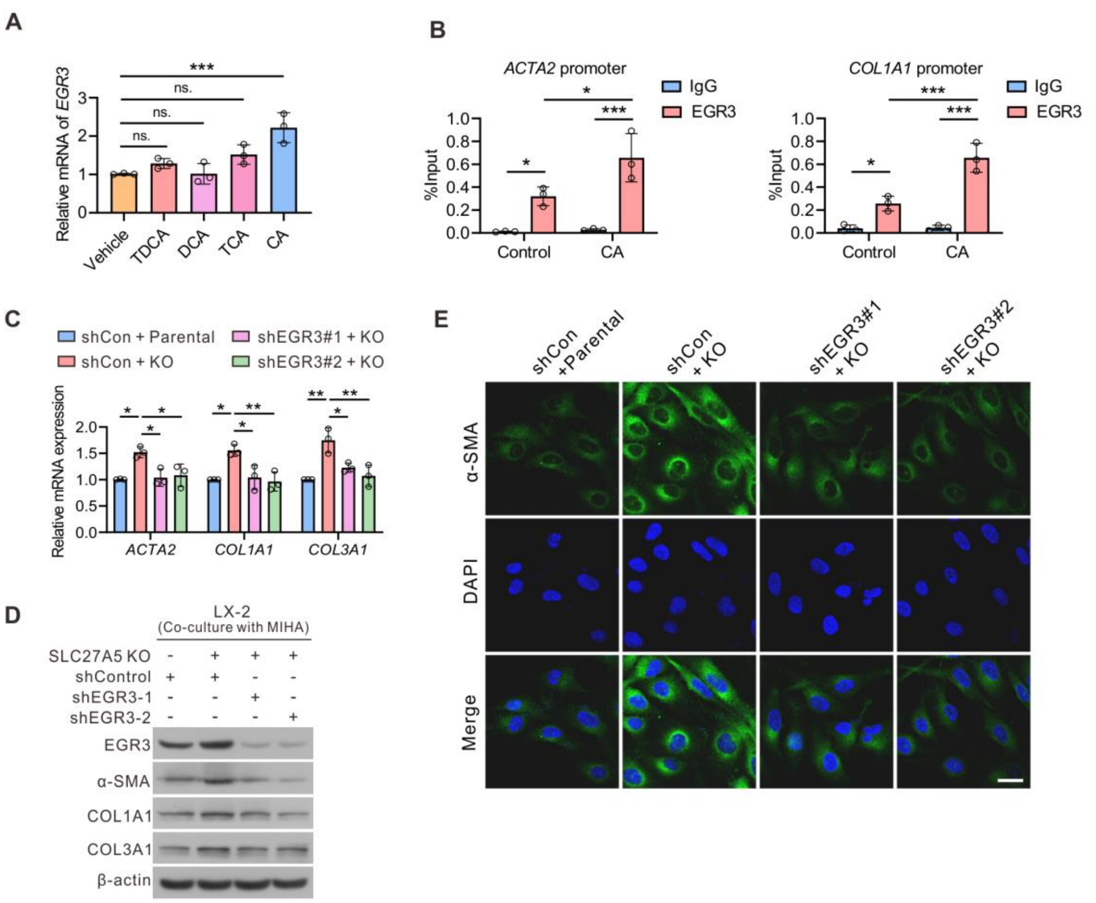

Figure S8. CA-triggered activation of HSC is dependent on EGR3.

(A) RT-qPCR analysis of *EGR3* expression in LX-2 cells treated with TDCA, DCA, TCA and CA at 50 μM (n=3 per group). (B) ChIP assay for EGR3's occupancy on the *ACTA2* and *COL1A1* gene promoter in LX-2 cells with or without CA (50 μM) treatment (n=3 per group). (C-E) LX-2 cells were transfected with Control (shCon) or shEGR3 plasmid and co-cultured with SLC27A5-KO MIHA cells (KO) for 48 hours. Expression of the fibrotic genes was analyzed by RT-qPCR (C), Western blotting (D) and immunofluorescence (E). Scale bar, 25 μm. Data are mean ± SEM. *$P < 0.05$, **$P < 0.01$, ns., not significant. Data in (A) and (C) were analyzed using one-way ANOVA with the Tukey's post hoc test. Data in (B) were analyzed using two-way ANOVA with Tukey's multiple comparisons test.

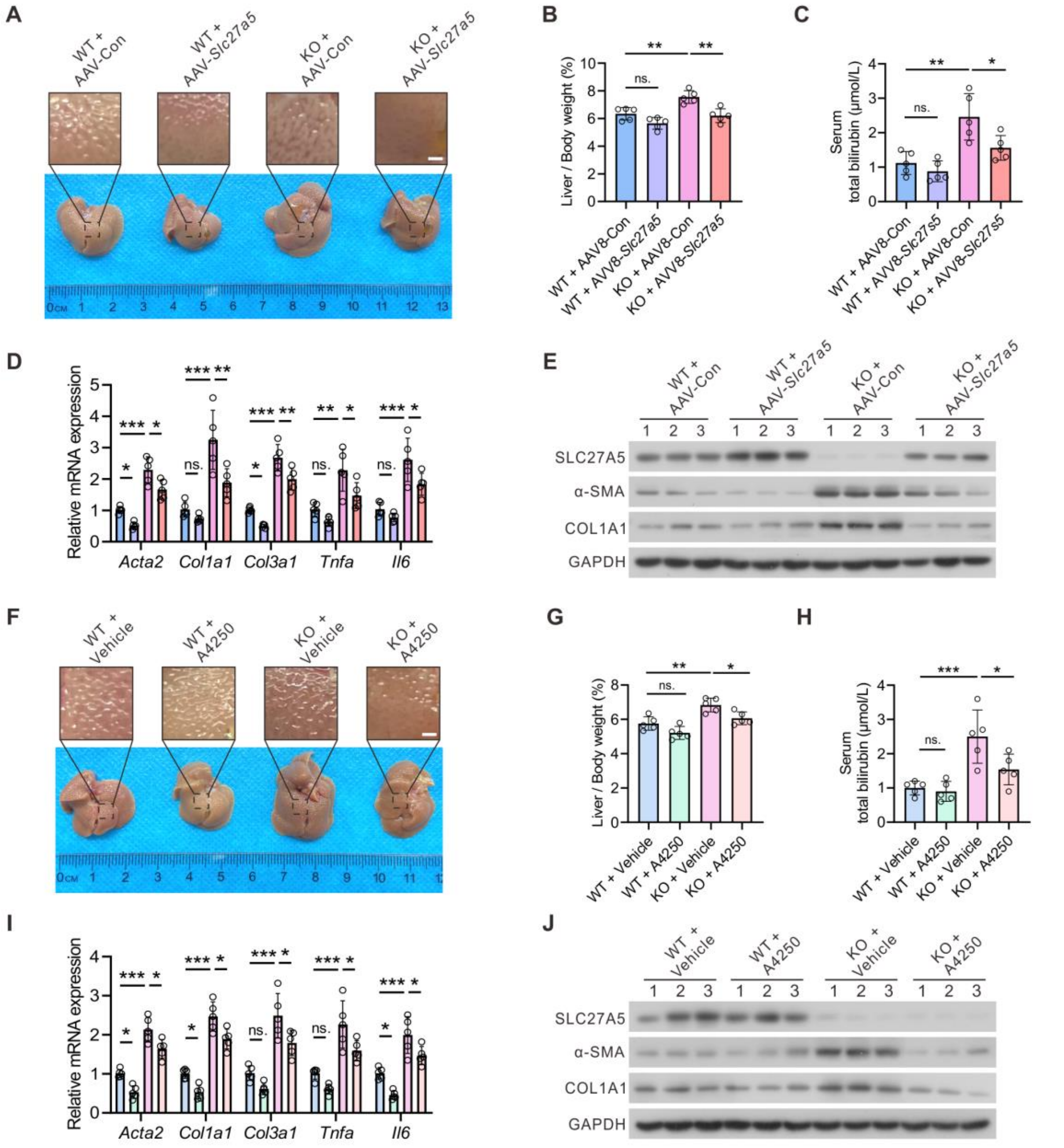

Figure S9. Overexpression of SLC27A5 or inhibition of intestinal bile acid absorption ameliorates CCl_4-induced liver fibrosis

(A-E) Eight-week-old male WT and *Slc27a5*$^{-/-}$ (KO) mice were subjected to the CCl_4 model and injected with AAV-Control (AVV-Con) or AAV-*Slc27a5* (n=5 per group). (F-J) Eight-week-old male WT and *Slc27a5*$^{-/-}$ (KO) mice were subjected to the CCl_4 model and treated with vehicle or A4250 by daily gavages (n=5 per group). (A, F) Representative morphology of livers. Scale bar, 1 mm. (B, G) Hepatomegaly in mice represented by the ratio of liver to body weight. (C, H) The serum levels of total

bilirubin were measured. (D, I) RT-qPCR analysis of *Acta2*, *Col1a1*, *Col3a1*, *Tnfa* and *Il6* expression in the mice livers. (E, J) Immunoblotting analysis of SLC27A5, α-SMA and COL1A1 expression in the mice livers. Data are mean ± SEM. $^{*}P < 0.05$, $^{**}P < 0.01$, $^{***}P < 0.001$, ns., not significant, one-way ANOVA with the Tukey's post hoc test.

Table S1. Clinical characteristics of participants in this study.

	Control	Cirrhosis	*P* value
N (male/female)	18 (11/7)	20 (16/4)	NS
Age (years)	55.33±8.79	59.55±8.73	NS
BMI (kg/m^2)	23.69±2.15	22.72±2.84	NS
ALB (g/l)	45.25±5.74	30.31±6.82	<0.001
ALT (U/l)	29.61±7.59	59.70±39.43	0.0037
AST (U/l)	30.06±7.91	81.20±70.67	0.0052
ALP (U/l)	63.28±14.92	197.15±134.61	<0.001
TBA (μmol/l)	5.32±1.82	66.37±68.01	<0.001
Compensation, N (%)	-	2 (10%)	-
Decompensation, N (%)	-	18 (90%)	-
Etiology			
Hepatitis B, N (%)	-	15 (75%)	-
Hepatitis C, N (%)	-	2 (10%)	-
Alcohol, N (%)	-	2 (10%)	-
Autoimmunity, N (%)	-	1 (5%)	-

BMI: body mass index; ALB: albumin; ALT: alanine transaminase; AST: aspartate transaminase; ALP: alkaline phosphatase; TBA: total bile acid. Data are mean ± SEM. Differences were analyzed by two-tailed Student's *t* test.

Table S2. Primer sequences used in this study.

qRT-PCR primers			
Species	Gene	Forward (5’-3’)	Reverse (5’-3’)
human	*SLC27A5*	AGCCCTGCCCTCTTCATCTA	GGTGGCTCCGAGATCTAAGC
human	*RUNX2*	CCGCCCCACGACAACC	TGACAGTAACCACAGTCCCATC
human	*ACTA2*	GGGGTGATGGTGGGAATG	GCAGGGTGGGATGCTCTT
human	*COL1A1*	GACGGCTCAGAGTCACCCA	GGAGACCACGAGGACCAGA
human	*COL3A1*	GCTCGGGGTAATGACGGT	AGGAATGCCAGCGGGAC
human	*TIMP1*	GCTTCTGGCATCCTGTTGTT	TGGTTGACTTCTGGTGTCCC
human	*MMP2*	TTTGACGGTAAGGACGGACTC	CCTGGAAGCGGAATGGAAAC
human	*CYP1B1*	CACTACTCGGAGCACTGGAAG	CGAAACACACGGCACTCAT
human	*EGR3*	TGCCAGGACAACATCATTAGC	TTGGAGTAGGGGGGTAGCG
human	*ESM1*	TGGATGGCATGAAGTGTGG	TTTTCCCGTCCCCCTGT
human	*TNFSF15*	AGATAAGCCAAGGGCACACC	GACTCTGGGATCAGCAGGAAT
human	*TTC36*	TCGGAGAGGAAGCAGAAAAG	TGGGCACGGTTGTTGTAGG
human	*TUBA8*	CTGACCACCCACACCACACT	CGTCAAAGCGGAGAGAAGC
human	*CTRB2*	AGACCAAGTACAACGCCAACA	AGGACACAATGCCCACCAG
human	*ACSBG1*	GGACGCAGAGGGCATTG	GGCACAGGGGGCACATT
human	*SNTG1*	TTGCTGTGGATGGGGTCTG	CCAGGAAGGTCGGGGAGTA
human	*ACTB*	AGGCCAACCGCGAGAAGATGACC	GAAGTCCAGGGCGACGTAGCAC
human	*GAPDH*	GAAATCCCATCACCATCTTCCAGG	GAGCCCCAGCCTTCTCCATG
mouse	*Runx2*	GTCCCAACTTCCTGTGCTCC	GAAACTCTTGCCTCGTCCG
mouse	*Hnf4a*	GGCAGTCAAGGCTCAGGA	CGCTAACTGCTGGGGATG
mouse	*Rxra*	CCGCCCTTCTCTGTCATCA	CAGGTGTAGGTCAGGTCTTTGC
mouse	*Rest*	AGCCTGTGAACGAGGGACC	CCCACTTGAGCCAATGCC

mouse	*Nr2c2*	GCTTCTGTGGAGCGTTTGC	CGGTGGTGCTTGTTGATGAT
mouse	*Slc27a5*	CACCCCCAGGGCTACGCT	CAGTGCTTGCCGCTCTAAA
mouse	*Acta2*	CCCTGAAGAGCATCCGACA	CATCTCCAGAGTCCAGCACAA
mouse	*Col1a1*	ACCCTGCCCGCACATG	CCCTCGCTTCCGTACTCG
mouse	*Col3a1*	GCCCACAGCCTTCTACACCT	TCCCGGATAGCCACCCA
mouse	*Timp1*	CCCAGAAATCAACGAGACCA	ACGCCAGGGAACCAAGAA
mouse	*VIM*	CAGCCTCTATTCCTCATCCCC	TGTAGTTGGCAAAGCGGTCAT
mouse	*Tnfa*	CCTGCCCCAAGGACACC	AGAGCAATGACTCCAAAGTAGACC
mouse	*Il6*	GTTGTGCAATGGCAATTCTGA	AAGGACTCTGGCTTTGTCTTTCT
mouse	*Il1b*	AAGCCTCGTGCTGTCGGA	CCATCTTCTTCTTTGGGTATTGC
mouse	*Ccl2*	TGTGCTGACCCCAAGAAGG	GGTGGTTGTGGAAAAGGTAGTG
mouse	*Adgre1*	CCAGTCAGATGATTCAGACGGAG	CAGTGCCACCAACAACACG
mouse	*Cyp7a1*	CCAGGGAGATGCTCTGTGTT	CGGGCTTTATGTGCGGTC
mouse	*Cyp7b1*	TTCTCTTTGCCGCCACC	TTTGGGTTTTTCGTTACATACTG
mouse	*Cyp8b1*	CCAAGCACGGGGATGTCT	GGTTGAGTTCCTCCAAGCCT
mouse	*Cyp27a1*	CTGGGGTGGACACGACATC	GTATTCTTGGGGAAGAGAAAGC
mouse	*Slc27a2*	CTGTTCCGAGACGAGACGC	TGGCACGAATGTTGTAGTTGAG
mouse	*Baat*	ATGAATAGCCCCTACCAAATCC	CCACCAGCACCTCCAAACA
mouse	*Actb*	CGTTCAATACCCCAGCCATG	GACCCCGTCACCAGAGTCC
mouse	*Gapdh*	CTCCCACTCTTCCACCTTCG	CCACCACCCTGTTGCTGTAG
Genotyping PCR Primers			
Species	Name	Forward (5’-3’)	Reverse (5’-3’)
mouse	*Slc27a5*-KO	GCCAGTGTGCTGATTGTGGATC	TTTGGTAAGGTCCAGGGTGG
mouse	*Slc27a5*-WT	AAGCTCACTTCTTGGTTACACAG	TTTGGTAAGGTCCAGGGTGG
Molecular Cloning Primers			

Species	Name	Forward (5’-3’)	Reverse (5’-3’)
human	pAdTrack-TO4-*RUNX2*	CGCGTCGACC ATGGCATCAAACAGCCTCTT	TGCTCTAGA TCAATATGGTCGCCAAACAGA
human	pGL3-*SLC27A5* (-2023/+366)	CCGCTCGAGGCCTCCCGCACAGTC	ACCAAGCTTCGGCTCAAGCATCCC
human	pGL3-*SLC27A5* (-1001/+182)	CGCCTCGAGGCAGGAAACAGTGAGGG	CCCAAGCTTCCAGCCCAAGTAGCAC
human	sh*RUNX2* #1	TGCACTATCCAGCCACCTTTACTTCAAGAGAGTAAAGGTGGCTGGATAGTGCTTTTTTC	TCGAGAAAAAAGCACTATCCAGCCACCTTTACTCTCTTGAAGTAAAGGTGGCTGGATAGTGCA
human	sh*RUNX2* #2	TGCACTTCGTCAGGATCCTATCTTCAAGAGAGATAGGATCCTGACGAAGTGCTTTTTTC	TCGAGAAAAAAGCACTTCGTCAGGATCCTATCTCTCTTGAAGATAGGATCCTGACGAAGTGCA
human	sh*EGR3*#1	TGCGACTCGGTAGTCCATTACATTCAAGAGATGTAATGGACTACCGAGTCGCTTTTTTC	TCGAGAAAAAAGCGACTCGGTAGTCCATTACATCTCTTGAATGTAATGGACTACCGAGTCGCA
human	sh*EGR3*#2	TGCACCAAAGCGCAGAGCTTGCTTCAAGAGAGCAAGCTCTGCGCTTTGGTGCTTTTTTC	TCGAGAAAAAAGCACCAAAGCGCAGAGCTTGCTCTCTTGAAGCAAGCTCTGCGCTTTGGTGCA
mouse	sh*Egr3*#1	TGCAACAAGACCGTGACCTACTTTCAAGAGAAGTAGGTCACGGTCTTGTTGCTTTTTTC	TCGAGAAAAAAGCAACAAGACCGTGACCTACTTCTCTTGAAAGTAGGTCACGGTCTTGTTGCA
mouse	sh*Egr3*#2	TGCTCCATTCCGGAACATAAGCTTCAAGAGAGCTTATGTTCCGGAATGGAGCTTTTTTC	TCGAGAAAAAAGCTCCATTCCGGAACATAAGCTCTCTTGAAGCTTATGTTCCGGAATGGAGCA
human	sg*SLC27A5*	CACCGCATCTACAAAGGTGTCAGG	AAACCCTGACACCTTTGTAGATGC
ChIP-qPCR Primers			
Species	Gene	Forward (5’-3’)	Reverse (5’-3’)
human	*SLC27A5*	CCTGTTGCTTTTCTGTTTGGC	GGCTGGCATCTAACTCCCT
human	*ACTA2*	TATTTCCTACGTCTGAGAACTGCC	TGGACAAGCCCTGACAAGC
human	*COL1A1*	AGGACTTTGGTGGGTTCAAGA	GACAGCAATGGAGGGATGG
